# Molecular differentiation of the *Murraya paniculata* Complex (Rutaceae: Aurantioideae: Aurantieae)

**DOI:** 10.1186/s12862-019-1555-4

**Published:** 2019-12-30

**Authors:** Chung Huy Nguyen, G. Andrew C. Beattie, Anthony M. Haigh, Inggit Puji Astuti, David J. Mabberley, Peter H. Weston, Paul Holford

**Affiliations:** 1 Plant Protection Research Institute, Phường Đức Thắng, Quận Bắc Từ Liêm, Hà Nội, Việt Nam; 2 School of Science, Western Sydney University, Locked Bag 1797, Penrith, NSW 2751 Australia; 3 Bogor Botanic Garden, Paledang, Tengah, Kota Bogor, Bogor, Jawa Barat 16122 Indonesia; 4 National Herbarium of New South Wales, Royal Botanic Garden, Mrs Macquaries Road, Sydney, NSW 2000 Australia; 5 Wadham College, University of Oxford, Oxford, UK; 6 Department of Biological Sciences, Macquarie University, Sydney, NSW 2109 Australia

**Keywords:** *Murraya*, Rutaceae, Phylogeny, Molecular dating, Monophyly

## Abstract

**Background:**

Orange jasmine has a complex nomenclatural history and is now known as *Murraya paniculata* (L.) Jack. Our interest in this common ornamental stemmed from the need to resolve its identity and the identities of closely related taxa as hosts of the pathogen ‘*Candidatus* Liberibacter asiaticus’ and its vector *Diaphorina citri*. Understanding these microbe-vector-plant relationships has been hampered by taxonomic confusion surrounding *Murraya* at both the generic and specific levels.

**Results:**

To resolve the taxonomic uncertainty, six regions of the maternally-inherited chloroplastal genome and part of the nuclear-encoded ITS region were amplified from 85 accessions of *Murraya* and *Merrillia* using the polymerase chain reaction (PCR). Clustering used maximum parsimony (MP), maximum likelihood (ML) and Bayesian inference (BI). Chronograms were produced for molecular dating, and to test the monophyly of *Murraya* rigorously, using selected accessions of *Murraya* and 26 accessions of the Rutaceae and Simarubaceae. Sequence data from the ITS and chloroplastal regions suggest that *Murraya paniculata* (sensu (Swingle WT and Reece CR, The Citrus Industry, p. 190–430, 1967)) can be separated into four distinct but morphologically somewhat cryptic taxa: *Murraya paniculata* (sensu (Mabberley DJ, Taxon 65:366–371, 2016)), *M. elongata*, *M. sumatrana* and *M. lucida*. In addition, *Murraya omphalocarpa* was identified as a putative hybrid of *M. paniculata* and *M. lucida* with two geographically isolated nothovarieties representing reciprocal crosses. *Murraya* is monophyletic, and molecular dating suggests that it diverged from *Merrillia* during the Miocene (23–5 Ma) with this *Murraya* group speciating and dispersing during the Middle Miocene onwards.

**Conclusions:**

The accessions from Asia and Australasia used in this study grouped into biogeographical regions that match herbarium specimen records for the taxa that suggest natural allopatric distributions with limited overlap and hybridity. *Murraya paniculata* has been distributed around the world as an ornamental plant. The division of the *Murraya paniculata* complex into four species with a rare hybrid also confirms morphological studies.

## Background

Orange jasmine, also known as orange jessamine, Chinese box and mock orange, has had a complex nomenclatural history [1]. Its name is now confirmed as *Murraya paniculata* (L.) Jack, the name most widely used in commerce, as a result of Mabberley’s [2] successful proposal to conserve a specimen of orange jasmine as the type of this name. It is best known as a common ornamental plant in tropical, subtropical and warm-temperate regions of the world and has diverse uses. In Indonesia and Malaysia, wood ascribed to orange jasmine is used for the hilts of daggers (kris or kreeses) [3–6], bark or leaf extracts are used in folk medicine for a wide range of purposes [7], and roots are a source of the anti-implantation indol alkaloid yuehchukene [8]. It has been used in the breeding of rootstocks for citrus, as it may be a source of tolerance to lime and nematodes [3, 9].

Our interest in orange jasmine stemmed from the need to resolve its identity and its status as a host of ‘*Candidatus* Liberibacter asiaticus’ (α-Proteobacteria), the pathogen that causes the most severe form of huanglongbing (HLB or ‘yellow shoot disease’ or citrus greening), a devastating, incurable disease of citrus [10], and *Diaphorina citri* Kuwayama (Hemiptera: Sternorrhyncha: Psylloidea), the primary and most widespread vector of the pathogen [11]. Various authors have suggested that orange jasmine can be a host of ‘*Candidatus* Liberibacter asiaticus’ [12–15] whilst others [16–18]) concluded that it is not [19]. Understanding this microbe-plant relationship has been hampered by taxonomic confusion surrounding *Murraya* at both generic and specific levels.

At the generic level, the question of whether *Murraya* should be circumscribed broadly to include the curry leaf ‘*Murraya koenigii* (L.) Spreng.’ and its close relatives, or more narrowly to exclude them has recently been resolved decisively by molecular systematic research [20–25] which has shown that curry leaf is *Bergera koenigii* L., as originally described. *Murraya* sensu lato is polyphyletic and the type of the genus; *M. paniculata,* is more closely related to *Merrillia* than to *Bergera*. Swingle and Reece [3] considered *Merrillia* to be related to an ancestor of *Murraya*; however, they placed *Murraya* and *Merrillia* in separate subtribes in the tribe Clauseneae. Recent molecular studies have placed *Merrillia* and *Murraya* (sect. *Murraya*) in the tribe Aurantieae [26], as confirmed by others [20–25].

At the species level, as discussed in detail by Mabberley [1, 2], taxonomic confusion of orange jasmine has existed from the mid-eighteenth century with its first description as *Camunium vulgare* by Rumphius [4] who ascribed a plate of an unrelated species to the description [2]. Additional confusion was introduced by Linneaus [27, 28] with his descriptions of *Murraya exotica* and *Chalcas paniculata* (*M. paniculata*). Because the Latin polynomials used to describe species in the eighteenth Century were necessarily short and published descriptions were not linked to type specimens, species concepts were rather broad [29] with, for example, one name being applied to what is now recognised as several different species in different genera or conversely, multiple names being applied to what is now recognised as one species. Moreover, interpretation of species was commonly based on brief descriptions [30] and illustrations, rather than examination of specimens and continued in this manner until some years after the introduction of the first International Rules of Botanical Nomenclature [29, 31]. Consequently, the lack of detailed distinguishing characters and type specimens may have persuaded subsequent authors to synonymise or subsume *M. exotica* with (in) *M. paniculata* or the reverse [3, 6]. Others have described them morphologically as two species [32], and some have used chemotaxonomic methods to distinguish these two species [8, 33]. Despite the 20 years of effort spent typifying Linnaean plant names [29], taxonomic confusion remains for the *Murraya paniculata* Complex. Resolution of this confusion is possible using molecular techniques both at the generic [24, 25] and at the species level [34].

From the beginning of research on HLB in Indonesia from 2003 to 2009, funded by the Australian Centre for International Agricultural Research, we observed differences among plants that suggested the presence of two morphologically distinct species of *Murraya* in Java. Here we report molecular differentiation of 82 accessions of plants of the genus *Murraya*, including plants that have been identified as *M. elongata* A.DC. ex Hook.f., *M. lucida* (G. Forst.) Mabb., *M. omphalocarpa* Hayata, *M. paniculata* (L.) Jack, *M. cyclopensis* Astuti & Rugayah and *M. sumatrana* Roxb. Our work is based on six regions of the maternally-inherited chloroplastal genome and the internal transcribed spacers (ITS) of nuclear ribosomal DNA region. We used *Merrillia caloxylon* (Ridl.) Swingle (Aurantieae) and two accessions from *Murraya* sect. *Bergera* (sensu [35, 36]), from the tribe Clauseneae, as outgroups. We used this data to address the question of whether the *M. paniculata* Complex consists of a single species, two species or may include additional cryptic species, as well as to determine the phylogeny of *Murraya*.

## Results

### Phylogeny derived from the six chloroplastal regions

The phylogenetic analysis of the individual chloroplastal regions did not show any topological incongruence between significantly supported components among the individual analyses (data not shown). However, before combining the chloroplastal data sets for further analysis, ILD tests were performed among all pairwise combinations of the following regions: *trnL-F*, *psbM-trnD*^GUC^ and *trnC*^*GCA*^*-ycf6.* These regions are representative of those with different nucleotide substitution models (F81 + G, F81, GTR, respectively). The tests returned *P* values of 1.0, 0.174, and 0.506 for *trnL-F* and *psbM-trnD*^GUC^, *trnL-F* and *trnC*^*GCA*^*-ycf6* and *psbM-trnD*^GUC^ and *trnC*^*GCA*^*-ycf6,* respectively. These results show that the sequences of chloroplastal regions are homogeneous and can legitimately be combined.

**Fig. 1:**
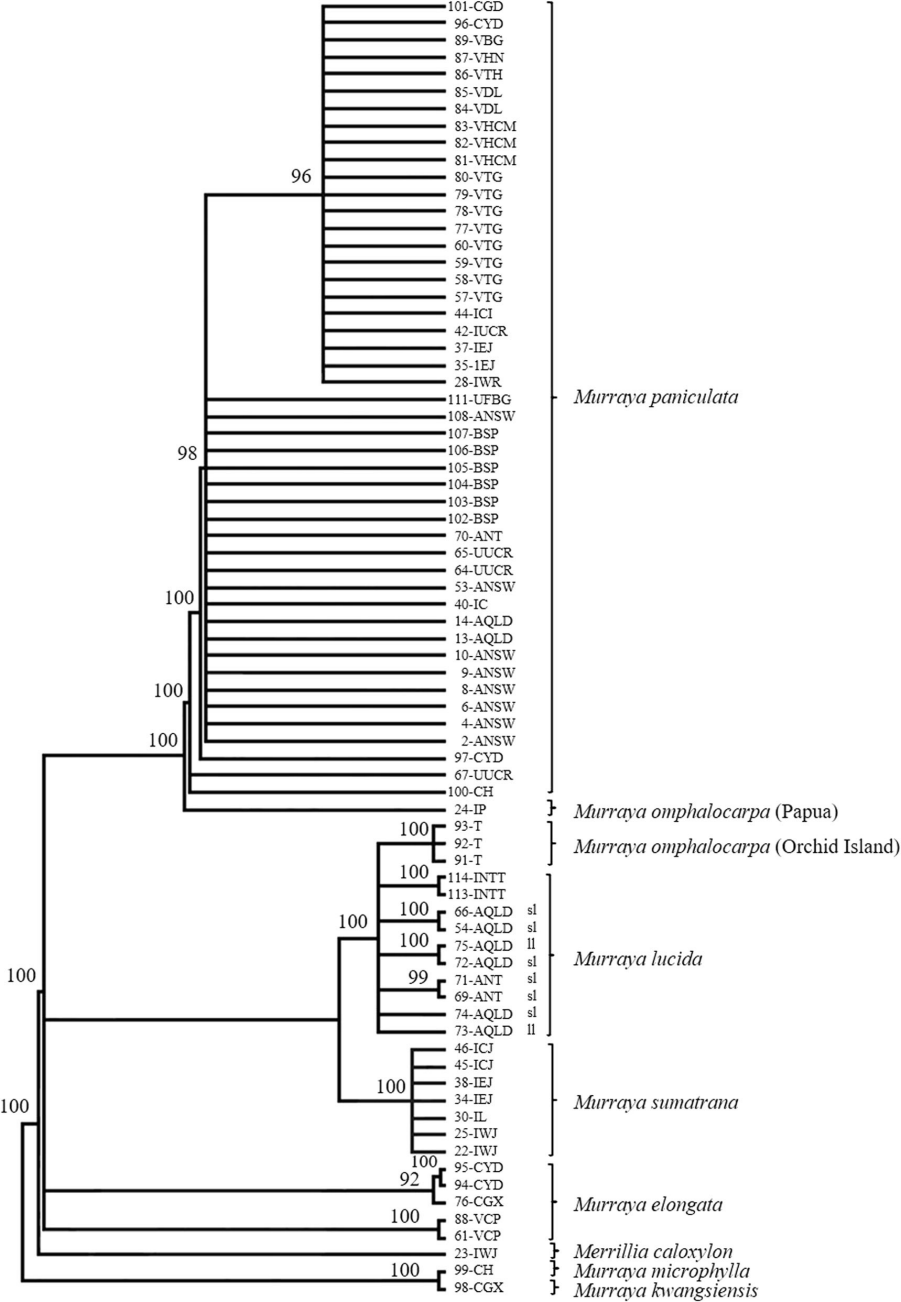
Bayesian inference tree based on the combined sequences of the six chloroplastal regions from accessions of *Murraya* and *Merrillia*. *Murraya kwangsiensis* and *M. microphylla* were used as the outgroup and posterior probabilities are shown above each branch. The model of nucleotide substitution used was GTR + G and the Markov chains were run for 5,000,000 generations (burnin = 1,250,000 generations). ‘sl’ small leaflet and ‘ll’ large leaflet forms of *Murraya lucida* from Australia

The length of the alignment of combined sequence data from the six different regions was 4627 bp of which 114 (2.46%) were phylogenetically informative characters (PICs). The majority rule consensus tree resulting from our Bayesian analysis of the combined plastid data set is shown in Fig. 1. The cladograms produced from parsimony and likelihood analyses of the data sets (with and without indels coded as binary characters) were topologically congruent with the Bayesian tree (see Fig. 1, Additional file 1: Figure S1).

In most of our chloroplastal trees, *Murraya* accessions formed clusters corresponding to the following taxa: *M. elongata*, *M. lucida*, *M. paniculata*, and *M. sumatrana*. In all analyses of the chloroplastal data, *Merrillia caloxylon* was placed as sister to the *Murraya* accessions and *M. kwangsiensis* (Huang) Huang and *M. microphylla* (Merr. et Chun) Swingle were not placed within the *Murraya* clusters. In most chloroplastal analyses, *M. lucida* clustered with *M. sumatrana*. Within this group, accessions of *M. lucida* clustered with accessions of *M. omphalocarpa* from Orchid Island to form a sub-group that was sister to the cluster of *M. sumatrana* accessions. Within the *M. omphalocarpa* – *M. lucida* cluster the accessions of *M. omphalocarpa* grouped together as did the accessions of *M. lucida* from Indonesia. However, the accessions of *M. lucida* from Australia were not resolved as a cluster, with three pairs of accessions and two single accessions forming a 7-way polytomy with *M. omphalocarpa* and *M. lucida* from Indonesia. In the MP analysis, all the *M. elongata* accessions grouped together, whereas using ML and BI these accessions separated into two unresolved sub-groups based on geographical origin with three accessions from China in one group and two from Việt Nam in the second.

In the *M. paniculata* group, in the MP, ML and BI analyses, accessions 24-IP from Papua, 67-UUCR from the University of California, Riverside, 100-CH from Hainan, China and 97-CYD from Yingde, China formed a basal paraphyletic grade. The remaining accessions cluster together in a 22-way polytomy within which 23 accessions group together as a sub-cluster. The ungrouped accessions in this polytomy are predominantly from Australia and Brazil, including the dwarf cultivar, *‘*Min-a-Min’ (70-ANT), whereas the sub-cluster predominantly contains accessions from Việt Nam and Indonesia. Other accessions of *M. paniculata* from China and the USA are distributed between the polytomy and the sub-cluster.

The chloroplastal sequences contain 42 phylogenetically informative indels (*psbM-trnD*^GUC^: 10; *trnL-F*: *6*; *trnC*^*GCA*^*-ycf6*: 9; *rps16*: 7; *matK-*5′*trnK*: 6; *rps4-trnT*: 4*)* and the cladogram derived from their analysis is shown in Additional file 1: Figure S2. The results show that all *Murraya paniculata* accessions form a single cluster that separates from all accessions of the other clusters including those of *M. omphalocarpa*. Within the *M. paniculata* cluster, two accessions from China (97-CYD and 100-CH) weakly group together but not with other accessions from China. Accessions 111-UFBG from Florida and 82-VHCM from Việt Nam also weakly group together but not with other *M. paniculata* accessions from these countries. Accession 24-IP from Papua did not group with the *M. paniculata* accessions. Among the *M. elongata*, *M. lucida*, and *M. sumatrana* accessions, there is little resolution, with any resolution occurring being based on their geographical origin. For example, the *M. sumatrana* accessions from Indonesia form a cluster, as do the *M. omphalocarpa* accessions from Taiwan and the *M. lucida* accessions from the Northern Territory. However, many accessions formed a large polytomy.

### Phylogeny derived from the ITS region

**Fig. 2:**
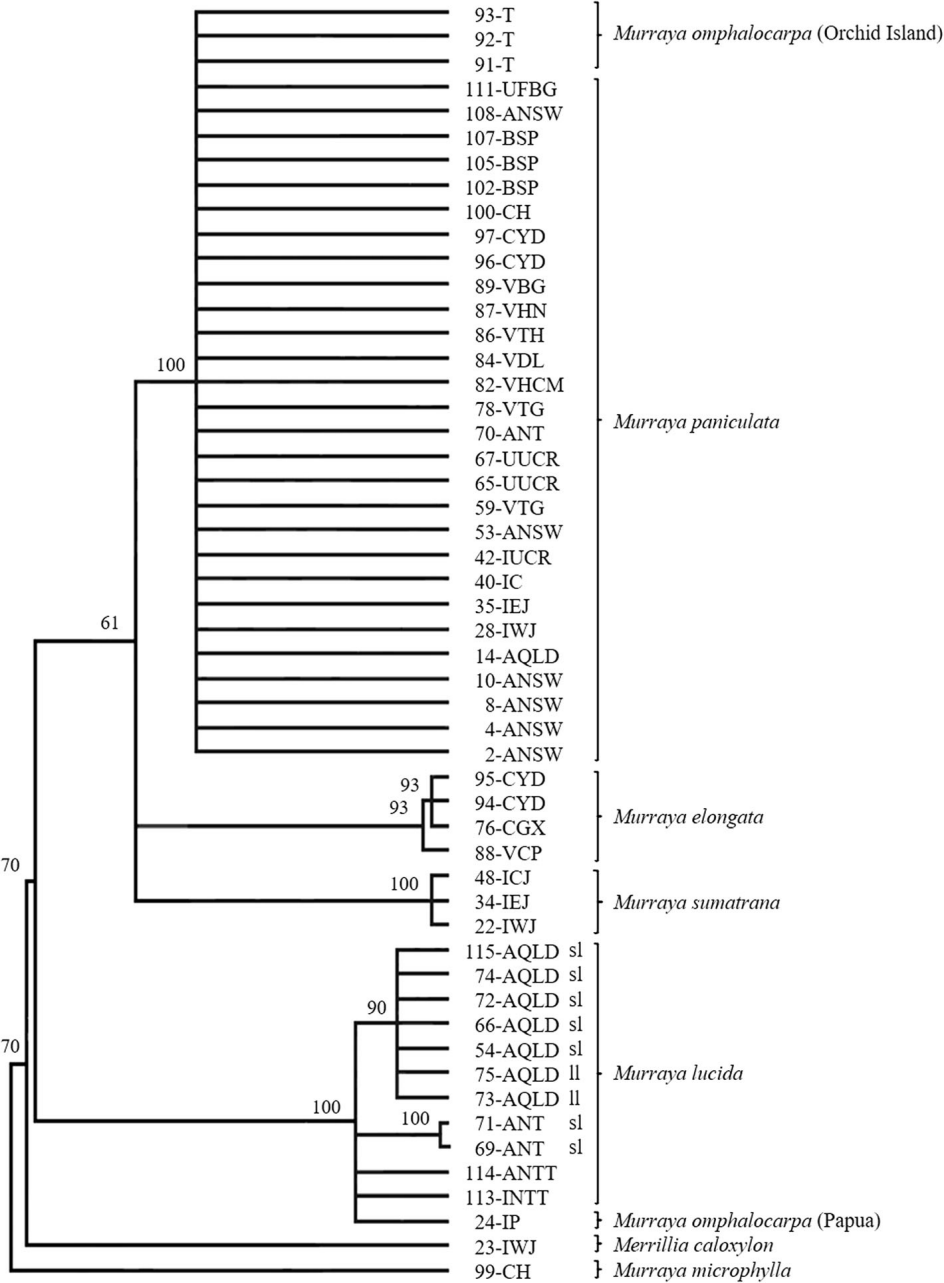
Bayesian inference tree based on the ITS sequences of accessions of *Murraya* and *Merrillia*. *Murraya microphylla* was used as the outgroup and posterior probabilities are shown above each branch. The model of nucleotide substitution used was GTR + G and the Markov chains were run for 5,000,000 generations with a sample frequency of 10 and a burnin of 1,250,000 generations. ‘sl’ small leaflet and ‘ll’ large leaflet forms of *Murraya lucida* from Australia

The phylogenetic relationships among accessions of *Murraya* and *Merrillia* were also examined using part of the nuclear rDNA ITS region. This analysis used 53 accessions of species of *Murraya* and *Merrillia*, which represent every clade and sub-clade found in the chloroplastal analyses. The sequence matrix consists of 625 nucleotides of which 51 sites (8.6%) were PICs. The trees produced by MP, ML and BI are identical; the tree produced by BI is shown in Fig. 2 (see Additional file 1: Figure S3) and consists of four main clusters containing: (1) accessions of *M. omphalocarpa* from Orchid Island plus all *M. paniculata* accessions including *‘*Min-a-Min’ but not 24-IP from Papua; (2) *M. elongata* accessions from China and Việt Nam; (3) *M. sumatrana* accessions from Java; (4) *M. lucida* from Indonesia, *M. lucida* from Australia*,* and 24-IP, with the accessions of *M. lucida* from the Northern Territory of Australia separating from those from Queensland. *Merrillia caloxylon* was resolved as the sister group of *Murraya and M. microphylla* was not placed among the *Murraya* clusters. The placement of 24-IP with *M. lucida* accessions and the placement of the accessions of *M. omphalocarpa* from Orchid Island with *M. paniculata* accessions are both strongly incongruent with their positions in the cpDNA trees.

### Phylogeny derived from combination of sequences of 6 chloroplastal genes and the ITS regio**n**

**Fig. 3:**
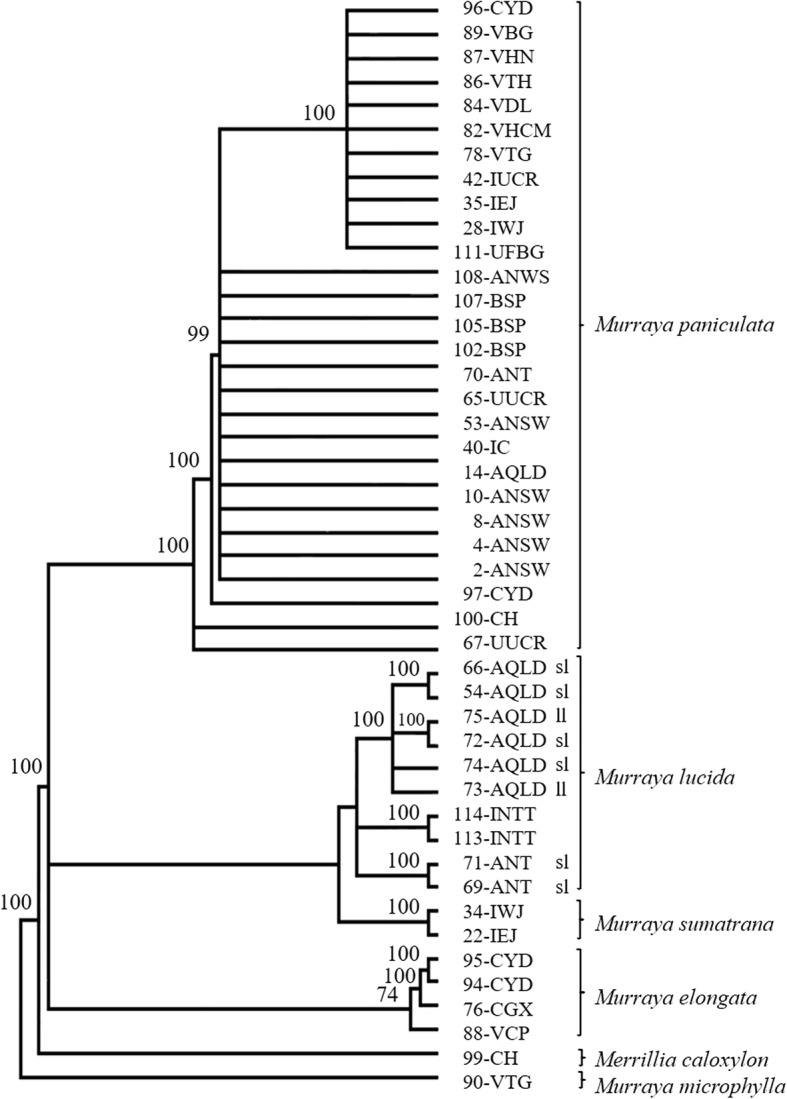
Bayesian inference tree based on the ITS region combined with 6 chloroplastal regions of accessions of *Murraya* and *Merrillia*. *Murraya microphylla* was used as the outgroup and posterior probabilities are shown above each branch. The model of nucleotide substitution used was GTR + G and the Markov chains were run for 600,000 generations with a sample frequency of 10 and a burnin of 150,000 generations. ‘sl’ small leaflet and ‘ll’ large leaflet forms of *Murraya lucida* from Australia

Before combining the sequences of chloroplastal and ITS regions for phylogenetic analysis, an incongruence length difference (ILD) test was performed between the data from these two regions from 51 accessions of *Murraya* and *Merrillia caloxylon*: this test returned a *P* value of 0.001, indicating that the data sets were heterogenous and should not be combined. When the ILD test was repeated with data from 24-IP and the *M. omphalocarpa* accessions from Orchid Island removed, it returned a P value of 0.03. Therefore, following this test, MP and BI analyses were performed on the combined chloroplastal and ITS data from 47 accessions of *Murraya* and *Merrillia*, but without the data from 24-IP and *Murraya omphalocarpa* from Orchid Island. This resulted in a sequence matrix consisting of 5218 nucleotides of which, 123 sites (2.35%) were PICs. The two trees resulting from these analyses are congruent and similar, and the tree resulting from Bayesian inference is presented in Fig. 3 (see Additional file 1: Figure S4).

In the cladograms produced by MP, ML and BI, *M. paniculata* accessions form a cluster that is clearly separated from the *M. elongata*, *M. lucida* and *M. sumatrana* accessions; the internal topology of the *M. paniculata* cluster is identical between these analyses. Using BI, all accessions of *M. elongata* form a single cluster separate from the other accessions, whereas using MP these accessions form two clusters based on geographic origin. With regards to the *M. lucida* and *M. sumatrana* accessions, a cluster is formed with the *M. sumatrana* accessions being sister to those of *M. lucida*. Using BI, the *M. lucida* accessions form a three-way polytomy consisting of two groups of *M. lucida* accessions from Australia, one from Queensland and the other from the Northern Territory, the third being those of *M. lucida* from Indonesia. However, using MP, the accessions of *M. lucida* from Indonesia are sister to the two Australian groups of *M. lucida.*

### Monophyly of *Murraya* and dating of divergence

**Fig. 4:**
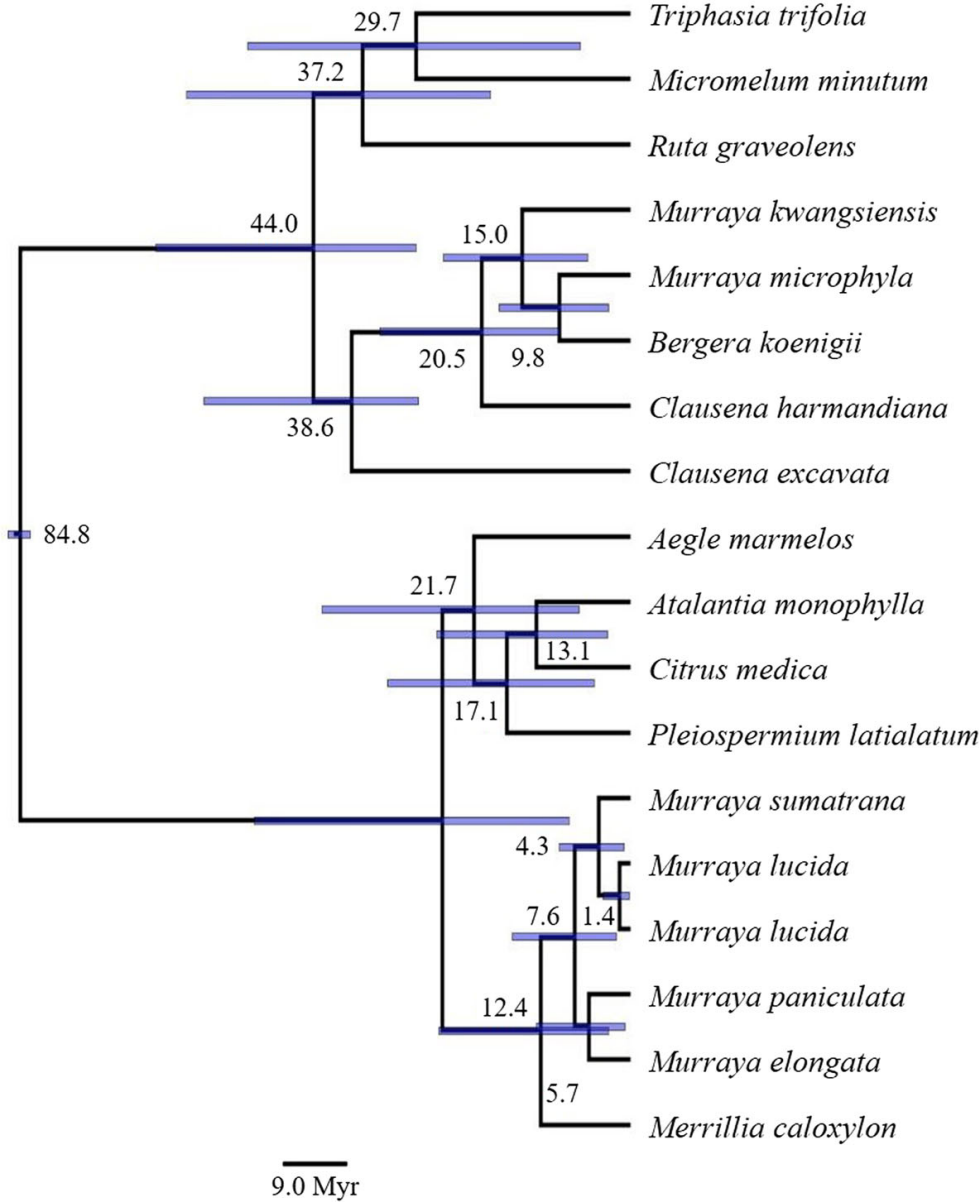
Maximum clade credibility tree produced using the BEAST suite of programs based on the combined sequences of five chloroplastal regions. The values next to the nodes are the ages (Ma). The bars represent the 95% highest posterior density

**Fig. 5:**
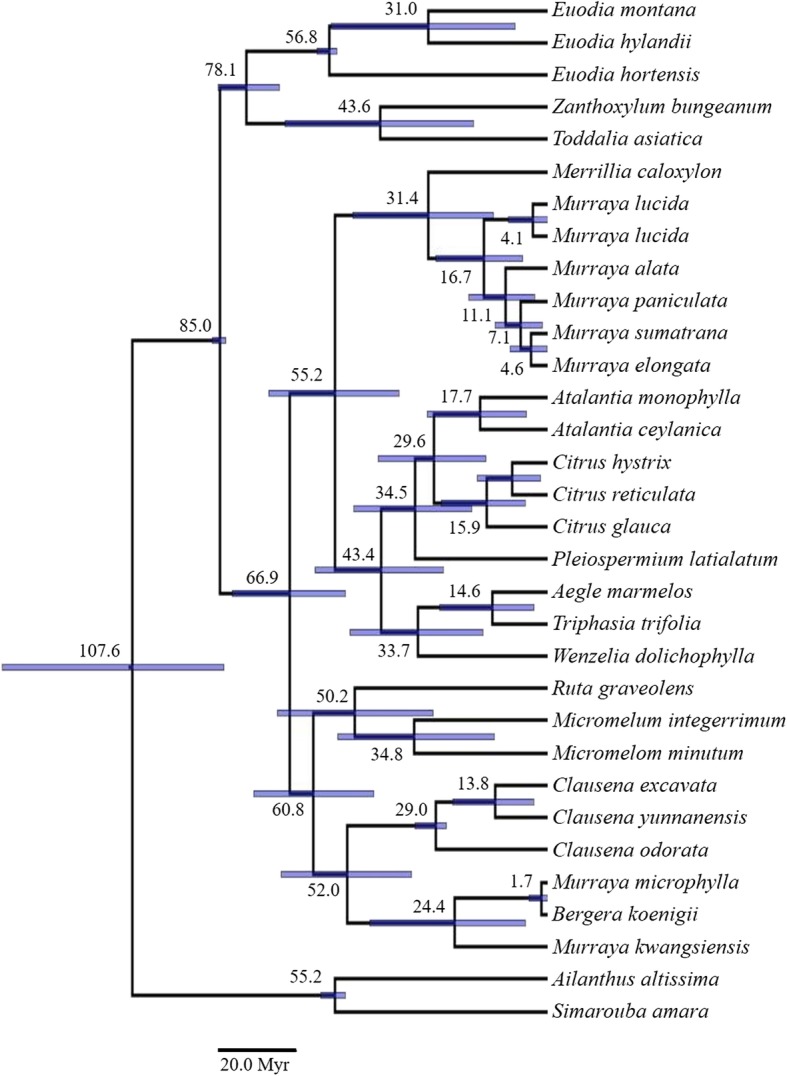
Maximum clade credibility tree produced using the BEAST suite of programs based on ITS regions. The values next to the nodes are the ages (Ma). The bars are the 95% highest posterior density

The ages of divergence and phylogenetic placement of accessions of *Murraya* and *Merrillia* was assessed against other species from the Rutaceae using chloroplastal (Fig. 4) or ITS sequences (Fig. 5). Both chronograms clearly show that the *Merrillia* and the four groups of *Murraya* accessions formed a single, distinct cluster that was separate from rutaceous accessions that have been placed within the subfamilies Toddalioideae and Aurantioideae (sensu [3]), in particular the accessions of *Murraya* were separate from those of *B. koenigii*, *M. kwangsiensis* and *M. microphylla* which clustered together. The analysis of the ITS data also shows that *Murraya alata* Drake (a southern Chinese and Indochinese species cultivated in the South China Botanical Gardens) grouped with the other *Murraya* accessions.

Dating of the divergence of these accessions differed between the analyses of the two data sets, with the ITS data giving older times for divergence than the chloroplastal data. The divergence between *Murraya* and *Merrillia* was estimated to have occurred 12.4 (95% HPD: 3.0–26.6) Ma from the chloroplastal data and 31.4 (14.3–50.7) Ma from the ITS data. The mean ages for divergence among the *Murraya* accessions was from 1.4–7.6 Ma according to the chloroplastal data and from 4.1–16.7 Ma from the ITS data; within each of these analyses, there is substantial overlap in the HPD intervals. Additionally, the dating of the divergence between the *M. paniculata* and *M. elongata* accessions in the analysis of the chloroplastal data of 5.7 (0.6–13.0) Ma was similar to that between the *M. paniculata* and *M. elongata* and *M. sumatrana* accessions in the ITS analysis of 7.1 (1.7–13.8) Ma.

### *Murraya omphalocarpa* putative hybrids

In the analysis of the six chloroplastal regions, the *M. omphalocarpa* accession, 24-IP, from Papua forms a clade sister to all *M. paniculata* accessions; whereas the *M. omphalocarpa* accessions from Orchid Island Taiwan form a clade that is sister to accessions of *M. lucida* (Fig. 1). In contrast, in the analysis of the ITS sequences, the *M. omphalocarpa* accessions from Orchid Island are part of the polytomy of *M. paniculata* accessions, but the *M. omphalocarpa* accession from Papua lies within the *M, lucida* accessions (Fig. 2). These results suggest that *M. omphalocarpa* is a putative natural hybrid between *M. paniculata* and *M. lucida* that has evolved by different crosses in each location. In Papua, the female parent was *M. paniculata*; whereas on Orchid Island the female parent was *M. lucida*.

## Discussion

This study has examined the phylogenetic relationships among wild and cultivated accessions of the *Murraya paniculata* Complex from mainland Asia, the Malay Archipelago, Australasia, and California, Florida and Brazil in the Americas. In all analyses, *M. paniculata* sensu stricto accessions formed a cluster separate from the other accessions and the taxonomic implication of this is discussed below. Also, in all analyses, accessions of the mainland *M. elongata* (*M.* ‘*asiatica*’ in Nguyen [37]) also formed a distinct cluster or clusters separate from all other accessions.

In the analysis of the chloroplastal regions, accessions of the *M. lucida* (*M. ovatifoliolata* in Nguyen [37]) and *M. sumatrana* (‘*M. paniculata’* in Nguyen [37]) groups formed a third cluster with the two groups forming sister sub-clusters. However, with the ITS data, accessions of *M. lucida* formed a cluster that was weakly supported as the sister group of the rest of the complex. Taken together, the sequence data from the ITS and chloroplastal regions suggest that, based on the sampling to date, *M. paniculata* (sensu [3]) can be separated into four distinct but morphologically somewhat cryptic taxa: *M. paniculata* (sensu [1]), *M. elongata*, *M. sumatrana* and *M. lucida* (syn. *M. heptaphylla* Span., *M. paniculata* var. *zollingeri* Tan. and *M. paniculata* var. *ovatifoliolata* Engl.). The recognition of four taxa is in concordance with a study of their morphology [37].

Swingle and Reece [3] placed *Murraya* and *Merrillia* in separate subtribes in tribe Clauseneae: Clauseninae and Merrilliinae. They considered *Merrillia* to be an abnormal member of Clauseneae and possibly related to ancestral forms of *Murraya*. Tanaka [38] and But et al. [35] proposed the division of *Murraya* (sensu lato) into sect. *Murraya* and sect. *Bergera.* More recent studies have moved *Merrillia* and *Murraya* (sect. *Murraya*) to tribe Aurantieae [26], and further studies [20–25] have confirmed this. Analysis of both the chloroplastal and ITS regions from accessions of the four groups of the *Murraya paniculata* Complex and *Merrillia* used in this study clearly show that *Murraya* is monophyletic and sister to *Merrillia caloxylon*. The accessions of *M. kwangsiensis*, *M. microphylla* and *B. koenigii* are more closely related to one other and to accessions of *Clausena* than to the accessions of the *Murraya paniculata* Complex. Our results support the disintegration of *Murraya* (sensu lato) as *Murraya* (sensu stricto) and *Bergera* with *Merrillia* and *Murraya* constituting subtribe Merrilliinae sensu [3], of tribe Aurantieae [26]. Further, *M. microphyla* and *M. kwangsiensis* do not belong within *Murraya* and should be transferred to *Bergera*.

Although the trees derived from chloroplastal data and those from the nuclear ITS region were largely in agreement, some incongruence was found. Incongruence between gene trees for comparable samples of taxa are not uncommon [39]. For example, Seelanan et al. [40] found such differences among members of the Gossypieae [Malvales: Malvaceae] as well as within the genus *Gossypium* L., and Barber et al. [41] showed incongruence between gene trees among 23 species of *Sideritis* Tourn. ex L. [Lamiales: Labiatae/Lamiaceae]. In rare, extreme cases (e.g. [42]) the majority of clusters in one gene tree are incongruent with those in another. In this study, with respect to the chloroplastal data, the accession 24-IP from Papua was always placed in the *M. paniculata* cluster, whilst using the ITS region, it grouped with *M. lucida* accessions. A second anomaly was the placement of the three accessions of *M. omphalocarpa* from Orchid Island. Using the chloroplastal data, this taxon grouped with *M. sumatrana* and *M. lucida* accessions, whilst in the analyses using the ITS region, it grouped within the *M. paniculata* cluster. These anomalies are the result of well supported phylogenies from the chloroplastal and ITS analyses and, as such, fit into the ‘hard incongruence’ category proposed by Seelanan et al. [40].

Wendel and Doyle [39] suggested that phylogenetic incongruence may occur due to technical issues such as insufficient data or taxon sampling. However, they also suggested that incongruence may reflect something interesting about the biology of the taxa under study and may be due to processes at various organisational levels, from the gene to organism to taxon levels. Two reasons often thought to cause incongruence are incomplete lineage sorting and introgressive hybridisation. With incomplete lineage sorting, an ancestral polymorphism in a gene or haplotype that was present before a speciation event is inherited by one or both resulting lineages when speciation occurs [43]. The allele/haplotype causing the anomaly may have evolved independently for some time before speciation has occurred. Introgressive hybridization occurs when genetically differentiated taxa interbreed, after which extensive backcrossing occurs. The time of divergence between an incongruent allele/haplotype resulting from hybridization and its most closely related allele/haplotype can be younger than the speciation event at which the parents of the hybrid diverged [44]. Hybridization is an important evolutionary mechanism in plants [45–47]. It has been estimated that 25% of plant species hybridize [48] and Rieseberg et al. [49] provide a list of ~ 90 species where incongruence between molecular markers is thought to be due to hybridization and introgression. Hybridization provides a simple explanation for the anomalous data found in this study, with accession 24-IP from Cycloop in Papua being formed from a hybridization event between *M. paniculata* as the female parent and *M. lucida* as the male parent. In contrast, *M. omphalocarpa* from Orchid Island, Taiwan, appears to result from hybridization occurring between *M. lucida* as the female parent and *M. paniculata* as the male parent. Tippery and Les [50] recently identified a natural hybrid of *Nymphoides* based on a similar hard incongruence between chloroplastal and ITS data.

Although differences occurred between the placement of taxa and the dates of divergence between the ITS and chloroplastal data, the molecular dating suggests that *Murraya* diverged from *Merrillia* during the Miocene (23–5 Ma) with the *Murraya paniculata* Complex speciating and dispersing during the Middle Miocene onwards. Pfeil and Crisp [22] give a more recent date for the divergence between *Merrillia* and *Murraya* namely 9.2 Ma. However, they used dates for the age of the Rutaceae of around 54–59 Ma—we used ~ 80 Ma for the age of the Rutaceae [51]. During the Early Miocene, the Australasian plate came into contact with the Sundaland margin of the Asian plate. The northward movement of the Australasian plate caused episodes of uplift and the accretion of various terranes, and the subduction beneath Indonesia would have caused widespread volcanism resulting in a chain of island arcs [52–56]). About 10 Ma, the gap between the Australian and Asian plates was at its narrowest and the areas of possible land were relatively extensive facilitating the movement of plants and animals [53].

Today, species of *Murraya* occur naturally in south and eastern mainland Asia, the Indonesian Archipelago, the Philippines and Australasia while *Merrillia caloxylon* is native to Thailand, Malaysia (Peninsular and Sabah) and Sumatra [57, 58]. The timing of speciation divergence and the current natural distribution of *Merrillia caloxylon*, *M. elongata*, *M. lucida* and *M. sumatrana* in Malesia, between mainland Asia and Australia, suggests that *Merrillia* and *Murraya* originated in Sundaland. The fruits of *Merrillia caloxylon* have a thick, leathery exocarp and are large (approximately 100 mm long × 80 mm in diameter [3]); that may have limited their dispersal. *Murraya* species on the other hand, have small fruits with seeds that can easily be dispersed by birds; seed-dispersal by birds has been proposed to account for ~ 40% of colonization events in various island groups [59].

The distribution of *Murraya* species parallels that found by Muellner et al. [60] for members of tribe Aglaieae (Sapindales: Meliaceae). These authors suggest that the ancestral area of this tribe group is in Sundaland and that dispersal was a major factor driving divergence. The biogeographic patterns found by Muellner et al. [60] suggest dispersal routes to Wallacea, to the Philippines and to India and Indochina during the Miocene and Pliocene (5–1.6 Ma). Other dispersals of plant species have been suggested during these periods. The meta-analysis of Crayn et al. [61] found similar patterns in 49 clusters that are distributed across Wallace’s line from SE Asia to Australia, with mean inferred ages of dispersal ranging from 33.2 to 1.0 Ma. They found that of the 44 clusters for which direction of dispersal could reasonably be inferred, 63%, involved dispersal from Sundaland to Australia and only 27% from Australia to Sundaland. Ninety percent of the disjunct clusters were found to have animal-dispersed propagules. Sniderman and Jordan [62] found an even greater disparity than did Crayn et al. [61] in the inferred direction of dispersal of disjunct tropical rainforest species, with 89% of species for which direction could reasonably be inferred dispersing from Sundaland to Australia, the same direction as we have inferred for *Murraya*.

The results of this study show that *M. paniculata,* as circumscribed by Swingle and Reece [3], comprises four species, and hybrids:

### *Murraya lucida*

This species is distributed in eastern and central islands of the Malay Archipelago and Australasia. It is most probably the plant illustrated, albeit inaccurately, as t. 17 of Rumphius’s [1, 4]. Herbarium amboinense and poorly described by him as *Camunium vulgare*, based on material from southern Sulawesi and the central and northern Maluku Islands. It is also most probably the plant on which Burman [63] based his *Chalcas camuneng*, and consequently Linnaeus [27, 28] his *Chalcas paniculata*. It resembles the annotated specimen (Burman Herbarium G00404451) Kleynhoff sent to Burman from the Botanical Garden in Batavia, Java, in which plants cultivated from the Indonesian archipelago were grown [64, 65]. Following Mabberley’s [1] recent retypification of the genus *Murraya* and synonymy of *M. heptaphylla* with *M. lucida* [66], *M. lucida* incorporates *M. paniculata* var. *zollingeri* in Nusa Tenggara, Sulawesi and Maluku regions of Indonesia, and islands of the Philippines. It also includes the small- and large-leafleted forms sensu Swingle and Reece [3] of *M. lucida* in north and north-eastern Australia, east to Vanuatu where it was recorded by Johann Reinhold Forster and his son Georg [sic] in 1780 during Cook’s second voyage and subsequently described as *Limonia lucida* by G. Forster [67].

### *Murraya paniculata*

*Murraya paniculata* was recently confirmed as the specific name for the species that includes cultivars of the tropical to warm temperate ornamental known as orange jasmine [1]. It is molecularly and morphologically distinctly different from accessions of the other taxa in our study. Our dating evidence suggests separation of this taxon from its closest relatives (either *M. elongata* or *M. elongata* + *M. sumatrana*) is older than divergence within *M. lucida*. Accessions from China were dispersed throughout the *M. paniculata* sub-clusters derived from the cpDNA and the ITS analyses, whereas those from other countries were restricted to certain nodes within a sub-cluster. The distributions of these accessions, and pre-1941 herbarium specimens from forested localities in China (IBK, IMDY, NAS, PE and SYS, most accessible through CVH) are consistent with an origin of the species in a region that now encompasses Guangxi and Guangdong in southern China, neighbouring areas in Fujian, Hainan, Taiwan, Yunnan, and possibly northern Việt Nam. Kong et al. [8] reported that accessions belonging to *M. paniculata* (‘*M. exotica’*) occurred primarily in maritime sites on red, acid soils in southern China (including Hainan) and northern Việt Nam.

According to Hockings [68], ‘Min-A-Min’, (#70-ANT), a dwarf variant of *Murraya* growing to 1 m in height, was selected from “*M. paniculata* var. *ovatifoliolata”* and propagated by cuttings. In this study, ‘Min-A-Min’ falls in the *M. paniculata* cluster. This accession has very small leaves, leaflets and flowers and the leaves of the plant examined and the image (Figure 38) of the plant in Hockings [68] from which ‘Min-A-Min’ was selected resemble those from other *M. paniculata* accessions. Therefore, it appears to be a form of *M. paniculata* and is not derived from *M. lucida*.

### *Murraya sumatrana*

*Murraya sumatrana* was introduced to India from Sumatra [69]. Jack [5] collected it in Sumatra and initially regarded it as Linnaeus’s *Chalcas paniculata* [6, 70], describing it as *M. paniculata*. The natural distribution of *M. sumatrana* appears to include the western (Indonesia, excluding New Guinea) and central (Borneo and the Philippines) islands of the Malay Archipelago. It is the plant called *M. paniculata* in studies funded by the Australian Centre for International Agricultural Research on huanglongbing and *Diaphorina citri* in Indonesia from 2003 to 2009; orange jasmine in those studies was called *M. exotica* [71]. Merrill [72] noted that Jack’s description was apparently based on material from Penang or Singapore, but this is unlikely. Hunter [73] noted that a young plant yet to ripen seeds in a garden in Penang was the only *M. paniculata* (‘*M. exotica’*) on the island, and the granite-maritime soils of Penang and Singapore do not accord with Jones’ [74] account of *M. sumatrana* (‘*M. paniculata’*) usually growing on rocky soils or limestone in Borneo. Moreover, in correspondence between Jack and Nathaniel Wallich, it is clear that Jack collected *M. sumatrana*, ‘a tree’ with ‘ovate acuminate leaves’ and ‘wood most highly valued for making handles of kresses’ in Sumatra [6, 75].

### *Murraya elongata*

*Murraya elongata* A. DC. ex Hook. f. described from Myanmar [30] was not mentioned by Swingle and Reece [3] either as a good species, or as a synonym of *M. paniculata*. Kurz [75, 76], Gamble [77] and Brandis [78] mention it, the latter noting that it was possibly a variety of *M. exotica*. Its distribution is associated with limestone karst hills and rocky terrains from Pakistan through India (including the Andaman Islands), Myanmar, Thailand, peninsular Malaysia (including the Langkawi islands), Laos, Việt Nam to southern China, with some overlap with what we consider to be the natural distribution of *M. paniculata* in southern China. Accessions of *M. paniculata* and *M. elongata* closely group with *M. alata*, another Indochinese-southeast China species recorded from Việt Nam, southwest Guangdong, southwest Guangxi, and southern Hainan [3, 32]. Based on our study, *M. paniculata* and *M. exotica*, as referred to by Kong et al. [8], Li et al. [33], and Huang [79], are *M. elongata* and *M. paniculata*, respectively.

Om [80] recorded the presence of *M. elongata* and *M. paniculata* in Bhutan during studies on HLB and psyllid species occurring on citrus. She recorded *Diaphorina citri* developing on mandarin (*Citrus reticulata* Blanco), *B. koenigii*, *M. paniculata* and *M. elongata*, the latter being the first record of the psyllid developing on a species of *Murraya* native in the Indian subcontinent. Hollis [81] hypothesised that the *Diaphorina citri* originated on the subcontinent in association with “a native species of *Murraya*”. Om’s [80] studies in Bhutan suggest that the original host of *D. citri* was, most likely, *B. koenigii*, and found no evidence to suggest that *M. elongata* is host of ‘*Candidatus* Liberibacter asiaticus’.

### *Murraya omphalocarpa* putative hybrids

Based on the hard incongruence between the chloroplastal and ITS analyses, our study determined that *M. omphalocarpa* (syn. *M. paniculata* var. *omphalocarpa* (Hay.) Tan., *M. cyclopensis*) is a putative hybrid between *M. paniculata* and *M. lucida* with accessions from Orchid Island, Taiwan, representing a cross between *M. paniculata* as the male parent while the accession from Mount Cycloop, in central northern Papua, represents the reciprocal cross. The Cycloop nothovariety may have resulted from a recent introduction of *M. paniculata* to the region. The Orchid Island nothovariety appears to occur naturally in southern Taiwan, Orchid Island, and northern Luzon in the Philippines. According to specimens we have studied, it occurs within the naturally overlapping distributions of *M. lucida* and *M. paniculata*. Thus, *M. omphalocarpa* may be a natural hybrid.

## Conclusion

The accessions from Asia and Australasia used in this study grouped into biogeographical regions that suggest natural allopatric distributions with limited overlap. *Murraya paniculata* (orange jasmine) has been distributed around the world. The division of the *Murraya paniculata* Complex into four species and a hybrid with two nothovarieties conforms with the morphological studies reported in Nguyen [37]. Wider collection of material is now required, particularly from the Philippines and the distinctive *Murraya gleniei* Thwaites ex Oliv. to ascertain distributional boundaries and any further consideration of taxon ranking. In addition, studies with a wider range of accessions and using other nuclear genes are necessary to test the parentage of the putative hybrids. To date, only *Murraya paniculata* has been shown to be a host of the ‘*Ca*. Liberibacter asiaticus’ and ‘*Ca*. Liberibacter americanus’ causing huanglongbing, and it appears to be a transient host of the pathogens [19, 82]. The host status of the other taxa urgently needs to be determined.

## Methods

### Plant materials and DNA extraction

Mature leaflets from plants were collected from the wild or from gardens, parks, bushland and germplasm collections in Australia, Brazil, China, Indonesia, Pakistan, Taiwan, the United States of America (USA), and Việt Nam (Additional file 1: Table S1). This resulted in a total of 85 accessions of *Murraya* and *Merrillia* that were used for molecular phylogenetic analysis. Total DNA from samples from Australia, China (63-CGD and 68-CGD), Indonesia, Việt Nam, and Florida (111-UFBG and 112-UFBG) was extracted from leaf material following the modified methods of Doyle and Doyle [83] and Warude et al. [84]. The DNA of accessions from Brazil and Taiwan was extracted using the method of Murray and Thomson [85] by our colleagues in those countries. The DNA of samples collected from the University of California Riverside (UCR), and from China accessions (76-CGX, 94-CYD, 95-CYD, 96-CYD, 97-CYD, 98-CGX, 99-CH, 100-CH, 101-CGD) were extracted using the DNeasy Plant Minikit (Qiagen) and the HP Plant DNA Kit (Omega Bio-Tek), respectively, following the manufacturers’ instructions.

### DNA amplification

Six different regions and spacers of the maternally-inherited chloroplastal genome and part of the nuclear-encoded ITS region (Table 1) were amplified from DNA extracts using the polymerase chain reaction (PCR). PCR was performed in 25 μL volumes using: *Taq* DNA polymerase (0.2 U) (New England Biolabs); 1 × Thermopol buffer or Thermopol II buffer (New England Biolabs); an equimolar mix of 0.2 mM dNTPs (Fisher Biotech); 0.4 μM each primer; and 5 μg acetylated bovine serum albumin. The cycling conditions and magnesium concentrations are given in Table 1.

**Table 1:** List of primer sequences and references used for molecular phylogenetic analyses and conditions for PCR

Target sequence	Forward and reverse primer names	5′ – 3′ primer sequence	Reference	Temperature (°C) and durations (sec) of denaturation, annealing and extension and total number of cycles	Magnesium concentration (mM)
*trnL-F*	c	CGA AAT CGG TAG ACG CTA CG	Taberlet et al. [86]	94, 60; 55, 60; 72, 120; 30	2
	f	ATT TGA ACT GGT GAC ACG AG	Taberlet et al. [86]		
*psbM-trnD*^GUC^spacer	trnD^GUC^R	GGG ATT GTA GYT CAA TTG GT	Shaw et al. [87]: modified from Demesure et al. [88]	94, 60; 55, 60; 72, 210; 35	2
	psbMF	AGC AAT AAA TGC RAG AAT ATT TAC TTC CAT	Shaw et al. [87]		
*rps16*	rpsF	GTG GTA GAA AGC AAC GTG CGA CTT	Oxelman et al. [89]	95, 30; 60, 60; 72, 120; 33	2
	rpsR2	TCG GGA TCG AAC ATC AAT TGC AAC	Oxelman et al. [89]		
*matK-*5′*trnK* spacer	matK6	TGG GTT GCT AAC TCA ATG G	Johnson and Soltis [90]	94, 60; 50, 60; 72, 90; 35	2
	matK5′R	GCA TAA ATA TAY TCC YGA AAR ATA AGT GG	Shaw et al. [87]		
*trnC*^*GCA*^*-ycf6* region	ycf6R	GCC CAA GCR AGA CTT ACT ATA TCC AT	Shaw et al. [87]	94, 60; 50, 60; 72, 210; 35	2
	trnC^GCA^F	CCA GTT CRA ATC YGG GTG	Shaw et al. [87]		
*rps4-trnT* spacer	trnT^UGU^R	AGG TTA GAG CAT CGC ATT TG	Shaw et al. [87]	92, 60; 55, 60; 72, 180; 30	2.5
	rps4R2	CTG TNA GWC CRT AAT GAA AAC G	Shaw et al. [87]		
ITS	ITS1	TCC GTA GGT GAA CCT GCG G	White et al. [91]	94, 90; 55, 70; 72, 90; 30	2
	ITS4	TCC TCC GCT TAT TGA TAT GC	White et al. [91]		

### DNA sequencing and sequence assembly

Successful amplifications were purified using the Wizard® SV Gel and PCR Clean-Up System (Promega) following the manufacturer’s instructions. The purified PCR products were quantified using a NanoDrop 1000 spectrophotometer (Thermo Fisher Scientific) and diluted to 50 ng/μL. Both strands of purified fragments were sequenced using the same primers as were used for amplification (Table 1) by automated sequencing using an Applied Biosystems 3730XL sequencer at Macrogen Inc. (908 World Meridian Venture Center, #60–24, Gasan-dong, Geumchun-gu, Seoul 153–781, Korea). DNA Baser (v. 2.91, Heracle BioSoft) was used to compile contigs. Sequences were placed in GenBank under the following accession numbers—ITS: MK020214–266; trnT: MK214118–198; trnC: MK170731–814; rps16: MK170646–730; trnLF: MK214199–281; trnD: MK170517–593; matK: MK170487–516 & MK170594–645.

### Phylogenetic analysis

Multiple sequence alignments were obtained using ClustalW [92] as implemented in BioEdit v. 5.0.6 [93]; each alignment was checked by eye. Aligned datasets were then analysed using PAUP* 4.0b10 [94] using the maximum parsimony (MP) optimality criterion. Parsimony analysis was performed using tree-bisection-reconnection branch swapping with a heuristic search with 1000 bootstrap replicates, holding one tree at each step during stepwise addition and with the steepest descent option not in effect. The MP analysis was performed for individual cpDNA chloroplastal regions, for the ITS region, for a combination of all chloroplastal regions and finally for the combined chloroplastal and ITS regions. For the analysis of individual sequences, gaps were treated as missing data and branches with a minimum length of zero were collapsed. The analyses of the combined chloroplastal sequences were based on two matrices, one including gaps coded only as missing characters and the other comprising this first data matrix with the addition of data from indels that were scored for presence or absence using the criteria of Simmons and Ochoterena [95]. In addition, a data matrix that consisted of the presence/absence data from the indels was also subjected to phylogenetic analysis.

The chloroplastal and nuclear ITS sequence data were also analysed using maximum likelihood as implemented in MEGA7 [96] and Bayesian inference as implemented in MrBayes v. 3.1 [97]. Appropriate models of evolution were also determined using MEGA7 prior to analysis. Before BI analysis, an appropriate nucleotide substitution model was identified using hierarchical likelihood ratio tests (hLRTs) implemented in MrModeltest v. 2 [98] for selection of the best-fit model. The Markov chain Monte Carlo simulations (MCMC) were started with 100,000 generations and were run until the standard deviation of split frequencies was below 0.01. At this stage, the number of generations (ngen) to reach this level was recorded and posterior probability values calculated using a sample frequency of 100. Additionally, analyses were rerun for the combined cpDNA and ITS data following partitioning and evolutionary model identification using PartitionFinder2 [99] using the greedy algorithm [100], then analysed using Bayesian inference in MrBayes with 1,000,000 MCMC simulations, a burnin of 250,000 and sample frequency of 1000. In BI, ML and MP analyses, *Merrillia* (Aurantieae) (accession 23-IJW), *Murraya kwangsiensis* (accession 98-CGX) and/or *M. microphylla* (accession 99-CH) were used as outgroup(s).

### Incongruence length difference (ILD) test

Because analyses of multiple data sets do not result in mutual agreement of phylogenetic relationships, ILD tests were performed using the partition homogeneity test as implemented in PAUP*. The ILD tests were conducted between those different chloroplastal data sets that were representative of the different nucleotide substitution models determined by MrModeltest. ILD tests were also performed between the combined chloroplastal data set and the ITS dataset. A second ILD test was conducted on these data sets with accessions 24-IP, 91-T, 92-T and 93-T excluded. These are accessions of *M. omphalocarpa* and are the ones that caused significant incongruence in the topology of trees derived from chloroplastal and ITS data.

### Testing the monophyly of *Murraya* and dating of divergence

The BEAST v. 1.6.1 package [101] was used to produce chronograms for molecular dating and to test the monophyly of *Murraya* rigorously by including a broad range of outgroup taxa. Two sets of sequence data were examined (Additional file 1: Table S2). Firstly, ITS sequences for selected accessions of *Murraya* and 26 accessions of the Rutaceae and Simaroubaceae were analysed using the age calibration points in Appelhans et al. [51] for *Clausena*, ‘*Euodia* 2’ and *Ailanthus* to define the priors for, respectively, the *Bergera/Clausena*, *Euodia/Toddalia/Zanthoxylum* and the *Simarouba/Ailanthus* crown nodes. A lognormal prior (mean = 1; s.e. = 0.3; offset = 82) was assigned to the Rutaceae using the age range obtained by Appelhans et al. [51] and the GTR + G model of sequence evolution, identified as appropriate by MrModetest, was used. Prior to analysis, RDP2 [102] was used to examine the sequence data for recombination; no recombination was found. Secondly, data from the five chloroplastal regions were analysed for *Murraya* accessions and 10 accessions of other genera of Rutaceae. The calibration data for *Clausena* were again used, the root height of the tree was defined with the lognormal prior for the Rutaceae as above, and there were separate partitions for each DNA region each assuming the HYK model of evolution, identified as appropriate by MrModeltest. For both analyses, an uncorrelated, relaxed clock model [103] assuming a lognormal distribution of rates and a randomly generated starting tree were used, and the tree priors were set to the birth-death process [104, 105]. Tracer v. 1.7.1 [106] was used to check for chain convergence, appropriate burnin values and effective sample sizes.

## Supplementary information

**Additional file 1 Table S1.** List of accessions of *Murraya and Merrillia* used for molecular phylogenetic analyses and the locations from which they were sourced. **Table S2.** GenBank accession numbers for the regions used to determine the monophyly and dating of divergence of the *Murraya* accessions. **Figure S1**. Phylogenetic analysis of the combined sequences of six chloroplastal regions from accessions of *Murraya* and *Merrillia*. **Figure S2.** 50% majority-rule bootstrap consensus tree based on the indels of six chloroplastal regions from accessions of *Murraya* and *Merrillia* derived from maximum parsimony analysis. **Figure S3**. Phylogenetic analysis by of the ITS regions of accessions of *Murraya*. **Figure S4**. Bayesian inference tree based on the 6 chloroplastal regions combined with the ITS region of accessions of *Murraya* and *Merrillia* following partitioning and model selection using PartitionFinder 2 (Lanfear et al. 2016) using the greedy algorthim (Lanfear et al. 2012).

## Data Availability

All sequences generated during this study have been uploaded to Genbank.

## References

[1] Mabberley DJ (2016). The typification of *Murraya*, *M. exotica*, and *M. paniculata* (Rutaceae): its significance for the world citrus industry. Taxon.

[2] Mabberley DJ (2016). (2433) proposal to conserve the name *Chalcas paniculata* (*Murraya paniculata*) (Rutaceae) with a conserved type. Taxon.

[3] Swingle WT, Reece CR, Reuther W, Webber HJ, Batchelor LD (1967). The botany of *Citrus* and its wild relatives. The Citrus Industry.

[4] Rumphius GE. Herbarium Amboinense, vol. 5. Amsterdam: M. Uytwerf after Amsterdam; 1747.

[5] Jack W (1820). Descriptions of Malayan plants. Malayan Miscellanies.

[6] Burkill IH (1935). A dictionary of economic products of the Malay peninsula.

[7] Aziz S, Sukari M, Rahmani M, Kitajima M, Aimi N, Ahpandi N (2010). Coumarins from *Murraya paniculata* (Rutaceae). Malaysian J Analytical Sci.

[8] Kong YC, Cheng KF, Ng KH, But PPH (1986). Qianli, Yu SX, Chang HT, Cambie RC, Kinoshita T, Kan WS et al: a chemotaxonomic division of *Murraya* based on the distribution of the alkaloids yuehchukene and girinimbine. Biochem Syst Ecol.

[9] Bitters W, Brusca J, Cole D (1964). The search for new citrus rootstocks. California Citrograph.

[10] Bové JM (2006). Huanglongbing: a destructive, newly-emerging, century-old disease of citrus. J Plant Pathol.

[11] Halbert SE, Manjunath KL (2004). Asian citrus psyllids (Sternorrhyncha : Psyllidae) and greening disease of citrus: a literature review and assessment of risk in Florida. Fla Entomol.

[12] Li T, Ke C. Detection detection of the bearing rate of *Liberobacter asiaticum* in the citrus psylla and its host plant *Murraya paniculata* by nested PCR. Acta Phytophylacica Sinica. 2002;29:31–5.

[13] Lopes SA. Huanglongbing in Brazil. In: International Workshop for the Prevention of Citrus Greening Disease in Severely Infected Areas, Ishigaki, Japan, 6–7 December 2006. Tokyo: Multilateral Research Network for Food and Agricultural Safety. Japanese Ministry of Agriculture, Forestry and Fisheries; 2006. p. 11-9.

[14] Zhou LJ, Gabriel DW, Duan YP, Halbert SE, Dixon WN (2007). First report of dodder transmission of Huanglongbing from naturally infected *Murraya paniculata* to *Citrus*. Plant Dis.

[15] Walter AJ, Duan Y, Hall DG (2012). Titers of ‘*Ca*. Liberibacter asiaticus’ in *Murraya paniculata* and *Murraya*-reared *Diaphorina citri* are much lower than in *Citrus* and *Citrus*-reared psyllids. HortScience.

[16] Miyakawa T (1980). Experimentally-induced symptoms and host range of citrus likubin (greening disease). Ann Phytopathol Soc Jpn.

[17] Garnier M, Bové JM. Citrus greening disease and the greening bacterium. In: Moreno P, da Graça JV, Timmer LW, editors. Proceedings of the Twelfth Conference of the International Organization of Citrus Virologists, New Delhi, India, 23-27 November 1992. Riverside: International Organization of Citrus Virologists, University of California, Riverside; 1993. p. 212-9.

[18] Dai K, Ikeshiri T, Matsuura T, Kimura S, Hamagami A, Fujiwara Y, Kobashigawa Y, Miyakuni S. Investigation of host range of *Candidatus* Liberibacter asiaticum—is *Murraya paniculata* a host plant of *Candidatus* L. asiaticum? Res Bull Plant Protection Serv(Japan). 2005;41:53–7.

[19] Cifuentes-Arenas Juan Camilo, Beattie George Andrew Charles, Peña Leandro, Lopes Silvio Aparecido (2019). Murraya paniculata and Swinglea glutinosa as Short-Term Transient Hosts of ‘Candidatus Liberibacter asiaticus’ and Implications for the Spread of Huanglongbing. Phytopathology™.

[20] de Araújo EF, de Queiroz LP, Machado MA. What is *Citrus*? Taxonomic implications from a study of cp-DNA evolution in the tribe Citreae (Rutaceae subfamily Aurantioideae). Org Divers Evol. 2003;3:55–62.

[21] Morton CM, Grant M, Blackmore S (2003). Phylogenetic relationships of the Aurantioideae inferred from chloroplast DNA sequence data. Am J Bot.

[22] Pfeil BE, Crisp MD (2008). The age and biogeography of *Citrus* and the orange subfamily (Rutaceae: Aurantioideae) in Australasia and New Caledonia. Am J Bot.

[23] Morton Cynthia M. (2009). Phylogenetic relationships of the Aurantioideae (Rutaceae) based on the nuclear ribosomal DNA ITS region and three noncoding chloroplast DNA regions, atpB-rbcL spacer, rps16, and trnL-trnF. Organisms Diversity & Evolution.

[24] Bayer RJ, Mabberley DJ, Morton C, Miller CH, Sharma IK, Pfeil BE, Rich S, Hitchcock R, Sykes S (2009). A molecular phylogeny of the orange subfamily (Rutaceae: Aurantioideae) using nine cpdna sequences. Am J Bot.

[25] Penjor Tshering, Anai Toyoaki, Nagano Yukio, Matsumoto Ryoji, Yamamoto Masashi (2010). Phylogenetic relationships of Citrus and its relatives based on rbcL gene sequences. Tree Genetics & Genomes.

[26] Samuel R, Ehrendorfer F, Chase MW, Greger H (2001). Phylogenetic analyses of Aurantioideae (Rutaceae) based on non-coding plastid DNA sequences and phytochemical features. Plant Biol.

[27] Linnaeus C (1767). Mantissa Plantarum. Generum editionis VI. Et Specierum editionis II.

[28] Linnaeus C (1771). Mantissa Plantarum. Altera. Generum editionis VI. Et Specierum editionis II. Regni animalis appendix.

[29] Jarvis C (2007). Order out of Chaos: Linnaean plant names and their types.

[30] Hooker JD. The Flora of British India, vol. 1. London: L Reeve; 1875.

[31] Briquet J. Règles internationales de la nomenclature botaniques adoptées par le Congrès International de Botanique de Vienne 1905. Jena: Gustav Fischer; 1906.

[32] Zhang DX, Hartley TG, Mabberley DJ, Wu ZY, Raven PH, Hong DY (2008). Rutaceae. Flora of China.

[33] Li Q, Zhu LF, But PPH, Kong YC, Chang HT, Waterman PG (1988). Monoterpene and sesquiterpene rich oils from the leaves of *Murraya* species - chemotaxonomic significance. Biochem Syst Ecol.

[34] Ito Y, Tanaka N, Barford AS, Bogner J, Li J, Yano O, Gale SW (2019). Molecular phylogenetic species deliminitation in the aquatic genus *Ottelia* (Hydrocharitaceae) reveals cryptic diversity within a widespread species. J Plant Res.

[35] But PPH, Kong YC, Ng KH, Chang HT, Li Q, Yu SX, Waterman PG (1986). A chemotaxonomic study of *Murraya* (Rutaceae) in China. Acta Phytotaxonomica Sinica.

[36] Mou FJ. Systematics of Clauseninae (Rutaceae). Beijing: Graduate School of the Chinese Academy of Sciences; 2009.

[37] Nguyen CH (2011). Circumscription of *Murraya* and *Merrillia* (Sapindales: Rutaceae: Aurantioideae) and susceptibility of species and forms to huanglongbing.

[38] Tanaka T (1929). *Chalcas*, a Linnean genus which includes many new types of Asiatic plants. J Soc Trop Agric.

[39] Wendel Jonathan F., Doyle Jeff J. (1998). Phylogenetic Incongruence: Window into Genome History and Molecular Evolution. Molecular Systematics of Plants II.

[40] Seelanan T, Schnabel A, Wendel JF (1997). Congruence and consensus in the cotton tribe (Malvaceae). Syst Bot.

[41] Barber JC, Finch CC, Francisco-Ortega J, Santos-Guerra A, Jansen RK. Hybridization in Macaronesian *Sideritis* (Lamiaceae): evidence from incongruence of multiple independent nuclear and chloroplast sequence datasets. Taxon. 2007;56(1):74–88.

[42] Barrett RA, Bayly MJ, Duretto MF, Forster PI, Ladiges PY, Cantrill DJ (2018). Phylogenetic analysis of *Zieria* (Rutaceae) in Australia and New Caledonia based on nuclear ribosomal DNA shows species polyphyly, divergent paralogues and incongruence with chloroplast DNA. Aust Syst Bot.

[43] Galtier N, Daubin V (2008). Dealing with incongruence in phylogenomic analyses. Philos Trans R Soc B Biol Sci.

[44] Joly S, Starr JR, Lewis WH, Bruneau A (2006). Polyploid and hybrid evolution in roses east of the Rocky Mountains. Am J Bot.

[45] Arnold ML, Hodges SA (1995). Are natural hybrids fit or unfit relative to their parents. Trends Ecol Evol.

[46] Arnold ML (1997). Natural hybridization and evolution.

[47] Raven P (1980). Hybridization and the nature of species in higher plants. Can Bot Assoc Bull.

[48] Mallet J (2005). Hybridization as an invasion of the genome. Trends Ecol Evol.

[49] Rieseberg LH, Whitton J, Linder CR (1996). Molecular marker incongruence in plant hybrid zones and phylogenetic trees. Acta Botanica Neerlandica.

[50] Tippery NP, Les DH (2011). Evidence for the hybrid origin of *Nymphoides montana* Aston (Menyanthaceae). Telopea.

[51] Appelhans MS, Kessler PJA, Smets E, Razafimandimbison SG, Janssens SB (2012). Age and historical biogeography of the pantropically distributed Spathelioideae (Rutaceae, Sapindales). J Biogeogr.

[52] Pigram CJ (1987). Terranes and the accretion history of the Papua New Guinea orogen. AGSO J Aust Geol Geophys.

[53] Hall R, Metcalfe I, Smith JMB, Morwood M, Davidson ID (2001). Cenozoic reconstructions of SE Asia and the SW Pacific: changing patterns of land and sea. Faunal and floral migrations and evolution in SE Asia–Australasia.

[54] Hall R (2002). Cenozoic geological and plate tectonic evolution of SE Asia and the SW Pacific: computer-based reconstructions, model and animations. J Asian Earth Sci.

[55] Sanmartin I, Ronquist F (2004). Southern hemisphere biogeography inferred by event-based models: plant versus animal patterns. Syst Biol.

[56] Lohman DJ, de Bruyn M, Page T, von Rintelen K, Hal R, Ng PKL, Shih HT, Carvalho GR, von Rintelen T (2011). Biogeography of the Indo-Australian archipelago. Ann Rev Ecol Evol Syst.

[57] Stone BC, Jones DT (1988). New and noteworthy Rutaceae: Aurantioideae from northern Borneo. Studies in Malesian Rutaceae, V. Proc Acad Natl Sci Phila.

[58] Lim TK (2012). *Merrillia caloxylon*, Edible and Non-Medicinal Plants, vol. 4, Fruits.

[59] Carlquist S, Keast A, Miller SE (1996). Plant dispersal and the origin of Pacific island floras. The origin and evolution of Pacific island biotas, New Guinea to eastern Polynesia: patterns and processes.

[60] Muellner AN, Pannell CM, Coleman A, Chase MW (2008). The origin and evolution of Indomalesian, Australasian and Pacific island biotas: insights from Aglaieae (Meliaceae, Sapindales). J Biogeogr.

[61] Crayn Darren M., Costion Craig, Harrington Mark G. (2014). The Sahul-Sunda floristic exchange: dated molecular phylogenies document Cenozoic intercontinental dispersal dynamics. Journal of Biogeography.

[62] Sniderman JMK, Jordan GJ (2011). Extent and timing of floristic exchange between Australian and Asian rain forests. J Biogeogr.

[63] Burman NL (1768). Flora Indica: cui accedit series zoophytorum indecorum, nec non Prodromus Florae Capensis.

[64] Florijn PJ (1985). Geschiedenis van de errste hortus medicus in Indië. Tijdschrift voor de Geschiedenis der Geneeskunde, Natuurwetenschappen, Wiskunde en Techniek.

[65] Florijn PJ (1987). Biographical notes about four plant collectors in Asia mentioned by NL Burman in his Flora Indica (1768). Taxon.

[66] Mabberley DJ (2017). Mabberley's plant-book: a portable dictionary of plants, their classification and uses.

[67] Forster G (1786). Florulae insularum australium prodromus.

[68] Hockings D (1998). Mock orange *Murraya paniculata* var. *ovatifoliolata* ‘Min–A–Min’. Plant Var J.

[69] Lindley J (1819). Edwards’s botanical register: consisting of coloured figures of exotic plants, cultivated in British gardens; with their history and mode of treatment.

[70] Burkill IH (1916). William Jack’s letters to Nathaniel Wallich, 1819-1821. J Straits Branch R Asian Soc.

[71] Beattie G, Holford P, Mabberley D, Haigh A, Bayer R, Broadbent P (2006). Aspects and insights of Australia-Asia collaborative research on huanglongbing. In: Proceedings of the international workshop for the prevention of citrus greening disease in severely infected areas.

[72] Merrill ED (1952). William Jack's genera and species of Malaysian plants. J Arnold Arboretum.

[73] Hunter W (1909). Plants of Prince of Wales Island. J Straits Branch R Asiatic Soc.

[74] Jones DT, Soepadmo E, Wong K (1995). Rutaceae. Tree Flora of Sabah and Sarawak.

[75] Kurz S (1874). Contributions towards a knowledge of the Burmese flora. J Asiatic Soc Bengal.

[76] Kurz S (1877). Forest Flora of British Burma.

[77] Gamble JS (1902). A manual of Indian timbers; an account of the growth, distribution, and uses of the trees and shrubs of India and Ceylon with descriptions of their wood-structure.

[78] Brandis D (1906). Indian trees: an account of trees, shrubs, woody climbers, bamboos and palms indigenous or commonly cultivated in the British Indian empire.

[79] Huang CC (1997). Flora Reipublicae Popularis Sinicae.

[80] Om N (2017). The roles of psyllids, host plants and environment in the aetiology of huanglongbing in Bhutan.

[81] Hollis D (1987). A new citrus-feeding psyllid from the Comoro Islands, with a review of the *Diaphorina amoena* species group (Homoptera). Syst Entomol.

[82] Lopes SA, Frare GF, Camargo LEA, Wulff NA, Teixeira DC, Bassanezi RB, Beattie GAC, Ayres AJ (2010). Liberibacters associated with orange jasmine in Brazil: incidence in urban areas and relatedness to citrus liberibacters. Plant Pathol.

[83] Doyle JJ, Doyle LL (1990). Isolation of plant DNA from fresh tissue. Focus.

[84] Warude D, Chavan P, Joshi K, Patwardhan B (2012). DNA isolation from fresh, dry plant samples with highly acidic tissue extracts. Plant Mol Biol Report.

[85] Murray MG, Thompson WF (1980). Rapid isolation of high molecular weight plant DNA. Nucleic Acids Res.

[86] Taberlet P, Gielly L, Pautou G, Bouvet J (1991). Universal primers for amplification of three non-coding regions of chloroplast DNA. Plant Mol Biol.

[87] Shaw J, Lickey EB, Beck JT, Farmer SB, Liu W, Miller J, Siripun KC, Winder CT, Schilling EE, Small RL (2005). The tortoise and the hare II: relative utility of 21 noncoding chloroplast DNA sequences for phylogenetic analysis. Am J Bot.

[88] Demesure B, Sodzi N, Petit RJ (1995). A set of universal primers for amplification of polymorphic non-coding regions of mitochondrial and chloroplast DNA in plants. Mol Ecol.

[89] Oxelman B, Liden M, Berglund D (1997). Chloroplast rps16 intron phylogeny of the tribe Sileneae (Caryophyllaceae). Plant Syst Evol.

[90] Johnson Leigh A., Soltis Douglas E. (1994). matK DNA Sequences and Phylogenetic Reconstruction in Saxifragaceae s. str. Systematic Botany.

[91] White T.J., Bruns T., Lee S., Taylor J. (1990). AMPLIFICATION AND DIRECT SEQUENCING OF FUNGAL RIBOSOMAL RNA GENES FOR PHYLOGENETICS. PCR Protocols.

[92] Thompson JD, Higgins DG, Gibson TJ (1994). Clustal-W - improving the sensitivity of progressive multiple sequence alignment through sequence weighting, position-specific gap penalties and weight matrix choice. Nucleic Acids Res.

[93] Hall TA (1999). BioEdit: a user-friendly biological sequence alignment editor and analysis program for windows 95/98/NT. Nucleic Acids Symp Ser.

[94] Swofford DL (2002). PAUP*—phylogenetic analysis using parsimony * and other methods beta version 40b10.

[95] Simmons MP, Ochoterena H (2000). Gaps as characters in sequence-based phylogenetic analyses. Syst Biol.

[96] Kumar S, Strecher G, Tamura K (2019). MEGA7: molecular evolutionary genetics analysis version 7.0 for bigger datasets. Mol Biol Evol.

[97] Ronquist F, Huelsenbeck JP (2003). MrBayes, v. 3: bayesian phylogenetic inference under mixed models. Bioinformatics.

[98] Nylander JAA (2004). MrModeltest v2. Program distributed by the author.

[99] Lanfear R, Calcott B, Ho S, Guindon S (2012). PartitionFinder: combined selection of partitioning schemes and substitution models for phylogenetic analyses. Mol Biol Evol.

[100] Rambaut Andrew, Drummond Alexei J, Xie Dong, Baele Guy, Suchard Marc A (2018). Posterior Summarization in Bayesian Phylogenetics Using Tracer 1.7. Systematic Biology.

[101] Drummond AJ, Rambaut A (2007). BEAST: Bayesian evolutionary analysis by sampling trees. BMC Evol Biol.

[102] Martin DP, Williamson C, Posada D (2005). RDP2: recombination detection and analysis from sequence alignments. Bioinformatics.

[103] Drummond AJ, Ho SY, Phillips MJ, Rambaut A (2006). Relaxed phylogenetics and dating with confidence. PLoS Biol.

[104] Yule GU (1925). A mathematical theory of evolution, based on the conclusions of Dr. J. C. Willis, F.R.S. Philos Trans R Soc B Biol Sci.

[105] Gernhard T (2008). The conditioned reconstructed process. J Theor Biol.

[106] Rambaut A, Drummong AJ, Xie W, Baele G, Suchard MA: Tracer: MCMC trace analysis tool v. 1.7.1 2003–2018.

