# Supplementary material for: Molecular differentiation of the Murraya paniculata Complex (Rutaceae: Aurantioideae: Aurantieae)
Source: BMC Evol Biol. 2019 Dec 30;19:236. doi: 10.1186/s12862-019-1555-4 (PMC6937641; doi:10.1186/s12862-019-1555-4)
Supplement: Supplementary file 1 — Additional file 1 Table S1. List of accessions of Murraya and Merrillia used for molecular phylogenetic analyses and the locations from which they were sourced. Table S2. GenBank accession numbers for the regions used to determine the monophyly and dating of divergence of the Murraya accessions. Figure S1. Phylogenetic analysis of the combined sequences of six chloroplastal regions from accessions of Murraya and Merrillia. Figure S2. 50% majority-rule bootstrap consensus tree based on the indels of six chloroplastal regions from accessions of Murraya and Merrillia derived from maximum parsimony analysis. Figure S3. Phylogenetic analysis by of the ITS regions of accessions of Murraya. Figure S4. Bayesian inference tree based on the 6 chloroplastal regions combined with the ITS region of accessions of Murraya and Merrillia following partitioning and model selection using PartitionFinder 2 (Lanfear et al. 2016) using the greedy algorthim (Lanfear et al. 2012). [file 12862_2019_1555_MOESM1_ESM.docx]

Additional file for Nguyen et al 2019 Molecular differentiation of *Murraya paniculata* Complex (Rutaceae: Aurantioideae: Aurantieae)

**Table S1.** List of accessions of *Murraya and Merrillia* used for molecular phylogenetic analyses and the locations from which they were sourced. The following abbreviations are used: Australia (A), New South Wales (NSW), Queensland (QLD), Northern Territory (NT); Brazil (B), São Paulo (SP); China (C), Yingde (YD), Guangdong (GD), Guangxi (GX), Hainan (H); Indonesia (I), West Java (WJ), Central Java (CJ), East Java (EJ), Lombok (L), Nusa Tenggara Timur (NTT), Papua (IP); Taiwan (T); United States of America (U), University of California, Riverside (UCR), Fairchild Botanic Garden (FBG); Việt Nam (V), Tien Giang (TG), Cuc Phuong National Park (CP), Ho Chi Minh city (HCM), Dac Lac (DL), Thanh Hoa (TH), Hà Nội (HN), Bac Giang (BG). Voucher numbers are given for pressed specimens lodged at the National Herbarium of New South Wales, Royal Botanic Garden, Sydney (NSW).

| **Accession Number** | **Source** | **Latitude and longitude** | **Taxon to which accession was assigned** | **Voucher Number** |
| --- | --- | --- | --- | --- |
|  | **Australia** |  |  |  |
| 2-ANSW | Richmond, NSW | 33.617°S, 150.750°E | *Murraya paniculata* | 822701 |
| 4-ANSW | Richmond, NSW | 33.617°S, 150.750°E | *Murraya paniculata* | 822702 |
| 6-ANSW | Windsor, NSW | 33.617°S, 150.817°E | *Murraya paniculata* | 822703 |
| 8-ANSW | Royal Botanic Garden, Sydney, NSW | 33.867°S, 151.217°E | *Murraya paniculata* |  |
| 9-ANSW | Government House, Sydney, NSW | 33.867°S, 151.217°E | *Murraya paniculata* |  |
| 10-ANSW | Richmond, NSW | 33.600°S, 150.767°E | *Murraya paniculata* | 822704 |
| 13-AQLD | Brisbane, QLD | 27.467°S, 152.967°E | *Murraya paniculata* | 822705 |
| 14-AQLD | Brisbane, QLD | 27.450°S, 152.983°E | *Murraya paniculata* | 822706 |
| 53-ANSW | Royal Botanic Garden, Sydney, NSW | 33.867°S, 151.217°E | *Murraya paniculata* | 822707 |
| 54-AQLD | Bundaberg, QLD | 24.850°S, 152.400°E | *Murraya lucida* ‘small leaflet’ | 822732 |
| 66-AQLD | Woongarra, QLD (ex University of California, Riverside) | 24.900°S, 152.400°E | *Murraya lucida* ‘small leaflet’ |  |
| 69-ANT | Haddon Head Beach, Blue Mud Bay, NT | 13.367°S, 135.717°E | *Murraya lucida* ‘small leaflet’ | 822733 |
| 70-ANT | Darwin, NT | 12.450°S, 130.833°E | *Murraya paniculata* (‘Min-a-Min’) |  |
| 71-ANT | Gove, NT | 12.163°S, 136.717°E | *Murraya lucida* ‘small leaflet’ |  |
| 72-AQLD | Mt Carbine, QLD | 16517°S, 145.150°E | *Murraya lucida* ‘small leaflet’ | 822734 |
| 73-AQLD | Cooktown-Mt Webb National Park, QLD | 15.067°S, 145.117°E | *Murraya lucida* ‘large leaflet’ | 822730 |
| 74-AQLD | Battle Camp, QLD | 15.283°S, 144.717°E | *Murraya lucida* ‘small leaflet’ | 822735 |
| 75-AQLD | Cairns, QLD | 16.867°S, 145.667°E | *Murraya lucida* ‘large leaflet’ | 822731 |
| 108-ANSW | Richmond, NSW | 33.617°S, 150.750°E | *Murraya paniculata* |  |
| 115-AQLD | Tondoon Botanic Gardens, Gladstone, QLD (via Royal Botanic Garden, Sydney) | 23.883°S, 151.250°E | *Murraya lucida* ‘small leaflet’ | 822736 |
|  | **Brazil** |  |  |  |
| 102-BSP | Capão Bonito, SP | 24.000°S, 48.333°W | *Murraya paniculata* |  |
| 103-BSP | Capão Bonito, SP | 24.000°S, 48.333°W | *Murraya paniculata* |  |
| 104-BSP | Botucatu, SP | 22.883°S, 48.450°W | *Murraya paniculata* |  |
| 105-BSP | Botucatu, SP | 22.883°S, 48.450°W | *Murraya paniculata* |  |
| 106-BSP | Araraquata, SP | 21.783°S, 48.167°W | *Murraya paniculata* |  |
| 107-BSP | Araraquata, SP | 21.783°S, 48.167°W | *Murraya paniculata* |  |
|  | **China** |  |  |  |
| 62‑CGD | South China Agricultural University, GD | 23.150°N, 113.333°E | *Murraya paniculata* |  |
| 63-CGD | South China Agricultural University, GD | 23.150°N, 113.333°E | *Murraya paniculata* |  |
| 68-CGD | South China Agricultural University, GD | 23.150°N, 113.333°E | *Murraya paniculata* |  |
| 76-CGX | Guangxi via South China Botanical Gardens |  | *Murraya elongata* | 822742 |
| 94-CYD | Pipashan, Yingde County, GD | 24.283°N, 113.350°E | *Murraya elongata* | 822739 |
| 95-CYD | Pipashan, Yingde County, GD | 24.300°N, 113.350°E | *Murraya elongata* | 822740 |
| 96-CYD | Pipashan, Yingde County, GD | 24.283°N, 113.350°E | *Murraya paniculata* | 822717 |
| 97-CYD | Hengshitang, Yingde County, GD | 24.400°N, 113.300°E | *Murraya paniculata* | 822718 |
| 98-CGX | Guangxi via South China Botanical Gardens in Guangzhou, GD |  | *Murraya kwangsiensis* | 822748 |
| 99-CH | Hainan via South China Botanical Gardens in Guangzhou, GD |  | *Murraya microphylla* | 822749 |
| 100-CH | Bawangling, Hainan, via South China Botanical Gardens in Guangzhou, GD | 19.117°N, 109.067°E | *Murraya paniculata* | 822719 |
| 101-CGD | South China Agricultural University, GD | 23.150°N, 113.333°E | *Murraya paniculata* | 822720 |
|  |  |  |  |  |
|  | **Indonesia** |  |  |  |
| 22-IWJ | Bogor Botanic Garden, WJ | 06.600°S, 106.800°E | *Murraya sumatrana* | 822723 |
| 23-IWJ | Bogor Botanic Garden, WJ | 06.600°S, 106.800°E | *Merrillia caloxylon* | 822747 |
| 24-IP | Bogor Botanic Garden (from Pegunungan Cycloop, Papua) | 02.500°S, 140.517°E | *Murraya omphalocarpa* (putative hybrid) | 822746 |
| 25-IWJ | Bogor Botanic Garden, WJ (from Merubetiri National Park, EJ) | 06.600°S, 106.800°E | *Murraya sumatrana* | 822724 |
| 27-IWJ | Bogor Botanic Garden, WJ | 06.600°S, 106.800°E | *Murraya paniculata* | 822709 |
| 28-IWJ | Bogor Botanic Garden, WJ | 06.600°S, 106.800°E | *Murraya paniculata* | 822710 |
| 30-IL | Bogor Botanic Garden, WJ (from Lombok, NTT) | 06.600°S, 106.800°E | *Murraya sumatrana* |  |
| 34-IEJ | Purwodadi Botanic Garden, EJ | 07.800°S, 112.733°E | *Murraya sumatrana* | 822725 |
| 35-IEJ | Purwodadi Botanic Garden, EJ | 07.800°S, 112.733°E | *Murraya paniculata* | 822711 |
| 37-IEJ | Purwodadi Botanic Garden, EJ | 07.800°S, 112.733°E | *Murraya paniculata* | 822712 |
| 38-IEJ | Purwodadi Botanic Garden, EJ | 07.800°S, 112.733°E | *Murraya sumatrana* | 822726 |
| 40-IC | Bayan, Purworejo, CJ (from China) | 07.717°S, 109.933E | *Murraya paniculata* | 822713 |
| 42-IUCR | Bayan, Purworejo, CJ (from UCR) | 07.717°S, 109.933E | *Murraya paniculata* | 822714 |
| 44-ICJ | Bayan, Purworejo, CJ | 07.717°S, 109.933E | *Murraya paniculata* | 822715 |
| 45-ICJ | Bayan, Purworejo, CJ | 07.717°S, 109.933E | *Murraya sumatrana* | 822727 |
| 46-ICJ | Universitas Gadjah Mada, Yogyakata, CJ | 07.776°S, 110.374°E | *Murraya sumatrana* | 822728 |
| 47-ICJ | Universitas Gadjah Mada, Yogyakata, CJ | 07.776°S, 110.374°E | *Murraya paniculata* | 822716 |
| 48-ICJ | Universitas Gadjah Mada, Yogyakata, CJ | 07.776°S, 110.374°E | *Murraya sumatrana* | 822729 |
| 51-ICJ | Universitas Gadjah Mada, Yogyakata, CJ | 07.776°S, 110.374°E | *Murraya paniculata* |  |
| 113-INTT | Kupang, NTT | 10.200°S, 123.600°E | *Murraya lucida* | 822737 |
| 114-INTT | Kupang, NTT | 10.200°S, 123.600°E | *Murraya lucida* | 822738 |
|  | **Taiwan** |  |  |  |
| 91‑T | Orchid Island, Taiwan | 22.033°N, 121.533°E | *Murraya omphalocarpa* (putative hybrid) | 822743 |
| 92-T | Orchid Island, Taiwan | 22.033°N, 121.533°E | *Murraya omphalocarpa* (putative hybrid) | 822744 |
| 93-T | Orchid Island, Taiwan | 22.033°N, 121.533°E | *Murraya omphalocarpa* (putative hybrid) | 822745 |
|  | **United States of America** |  |  |  |
| 64-UUCR | University of California, Riverside | 33.967°N, 117.333°W | *Murraya paniculata* |  |
| 65-UUCR | University of California, Riverside | 33.967°N, 117.333°W | *Murraya paniculata* |  |
| 67-UUCR | University of California, Riverside | 33.967°N, 117.333°W | *Murraya paniculata* |  |
| 111-UFBG | Fairchild Botanic Garden, Florida | 25.683°N, 80.283°W | *Murraya paniculata* | 822721 |
| 112-UFBG | Fairchild Botanic Garden, Florida | 25.683°N, 80.283°W | *Murraya paniculata* | 822722 |
|  | **Việt Nam** |  |  |  |
| 57-VTG | Chau Thanh, Tien Giang | 10.400°N, 106.283°E | *Murraya paniculata* |  |
| 58-VTG | Chau Thanh, Tien Giang | 10.400°N, 106.283°E | *Murraya paniculata* |  |
| 59-VTG | Chau Thanh, Tien Giang | 10.400°N, 106.283°E | *Murraya paniculata* |  |
| 60-VTG | Chau Thanh, Tien Giang | 10.400°N, 106.283°E | *Murraya paniculata* |  |
| 61-VCP | Cuc Phuong National Park, Ninh Binh | 20.250°N, 105.700°E | *Murraya elongata* |  |
| 77-VTG | Cai Lay, Tien Giang | 10.417°N, 106.117°E | *Murraya paniculata* |  |
| 78-VTG | Cai Lay, Tien Giang | 10.417°N, 106.117°E | *Murraya paniculata* |  |
| 79-VTG | Cai Lay, Tien Giang | 10.350°N, 106.367°E | *Murraya paniculata* |  |
| 80-VTG | Chau Thanh, Tien Giang | 10.400°N, 106.283°E | *Murraya paniculata* |  |
| 81-VHCM | Dam Sen Park, Ho Chi Minh City | 10.765°N, 106.640°E | *Murraya paniculata* |  |
| 82-VHCM | Dam Sen Park, Ho Chi Minh City | 10.765°N, 106.640°E | *Murraya paniculata* |  |
| 83-VHCM | Tao Dan Park, Ho Chi Minh City | 10.775°N, 106.700°E | *Murraya paniculata* |  |
| 84-VDL | Buon Ma Thuot, Dac Lak | 12.666°N, 108.033°E | *Murraya paniculata* |  |
| 85-VDL | Buon Ma Thuot, Dac Lak | 12.666°N, 108.033°E | *Murraya paniculata* |  |
| 86-VTH | Dong Ve, Thanh Hoa | 19.783°N, 105.783°E | *Murraya paniculata* |  |
| 87-VHN | Bach Thao Park, Hà Nội | 21.033°N, 105.833°E | *Murraya paniculata* |  |
| 88-VCP | Cuc Phuong National Park, Ninh Binh | 20.250°N, 105.700°E | *Murraya elongata* | 822741 |
| 89-VBG | Việt Yen, Bac Giang | 21.283^o^N, 106.083^o^E | *Murraya paniculata* |  |

**Table S2.** GenBank accession numbers for the regions used to determine the monophyly and dating of divergence of the *Murraya* accessions. The subtribes Balsamocitrinae, Citrineae and Triphasiinae of the Aurantieae (Citreae) and Clauseninae and Micromelinae of the Clauseneae within the Aurantioideae are based on Swingle and Reece (1967), and the *Euodia* and *Ruta* Alliances within the Rutoideae of Kubitzki et al. (2011).

| **Species** | **ITS** | ***matK-*5′*trnK* spacer** | ***rps16*** | ***psbM-trnD*^GUC^ spacer** | ***trnL-F*** | ***rps4-trnT* spacer** |
| --- | --- | --- | --- | --- | --- | --- |
| *Aegle marmelos* (L.) Corr. Serr.  Balsamocitrinae | FJ434169 | EF138836 | AY295268 | EF164808 | AY295294 | EF134628 |
| *Ailanthus altissima* (Mill.) Swingle  Simaroubaceae | KM051450 |  |  |  |  |  |
| *Atalantia* *ceylanica* (Arn.) Oliv.  Citrinae | AB456053 |  |  |  |  |  |
| *Atalantia monophylla* (L.) DC.  Citrinae | GQ225867 | EF138841 | EF126570 | EF164813 | EF126636 | EF134633 |
| *Citrus glauca* (Lindl.) Burkill  Citrinae | FJ434161 |  |  |  |  |  |
| *Bergera koenigii* L.  Clauseninae | FJ434147 | EF138843 | EF138843 | EF126636 | EF134635 | EF134635 |
| *Citrus medica* L.  Citrinae | GQ225849 | EF138871 | KJ364715 | EF164843 | EF126665 | EF134663 |
| *Citrus reticulata* Blanco  Citrinae | JN661209 |  |  |  |  |  |
| *Clausena excavata* Burm. f.  Clauseninae | JX144188 | KF159531 | AF320260 | EF164853 | JX144230 | EF134673 |
| *Clausena harmandiana* (Pierre) Guillaumin  Clauseninae |  | EF138882 | EF126608 | EF164854 | EF126675 | EF134674 |
| *Clausena odorata* C.C.Huang  Clauseninae | JX144192 |  |  |  |  |  |
| *Clausena yunnanensis* C.C.Huang  Clauseninae | JX144193 |  |  |  |  |  |
| *Euodia hortensis* J.R. Forst. and G. Forst.  Euodia Alliance | HG002399 |  |  |  |  |  |
| *Euodia hylandii* T.G. Hartley  Euodia Alliance | HG971326 |  |  |  |  |  |
| *Euodia montana* T.G. Hartley  Euodia Alliance | HG971327 |  |  |  |  |  |
| *Micromelum integerrimum* (Buch.-Ham. ex DC.) Wight and Arn. ex M. Roem.  Micromelinae | JX144208 |  |  |  |  |  |
| *Micromelum minutum* (G. Forst.) Wight and Arn.  Micromelinae | FJ434148 | AB762396 | AF320266 | EF164876 | EF126691 | EF134696 |
| *Murraya alata* Drake  Clauseninae | JX144209 |  |  |  |  |  |
| *Pleiospermium latialatum* Swingle  Citrinae | FJ434157 | EF138913 | EF126628 | EF164885 | EF126697 | EF134705 |
| *Ruta graveolens* L.  *Ruta* Alliance (Ruteae) | JQ230976 | EF489057 | EU853765 | EF164887 | AY295275 | EF134707 |
| *Simarouba amara* Aubl.  Simaroubaceae | DQ787413 |  |  |  |  |  |
| *Toddalia asiatica* (L.) Lam.  *Euodia* Alliance | HG004786 |  |  |  |  |  |
| *Triphasia trifolia* (Burm. f.) P. Wilson  Triphasiinae | JX144220 | AB762386 | AY295271 | EF164893 | JX144266 | EF134713 |
| *Wenzelia dolichophylla* (Lauterb. and K. Schum.) Tan.  Triphasiinae | FJ434150 |  |  |  |  |  |


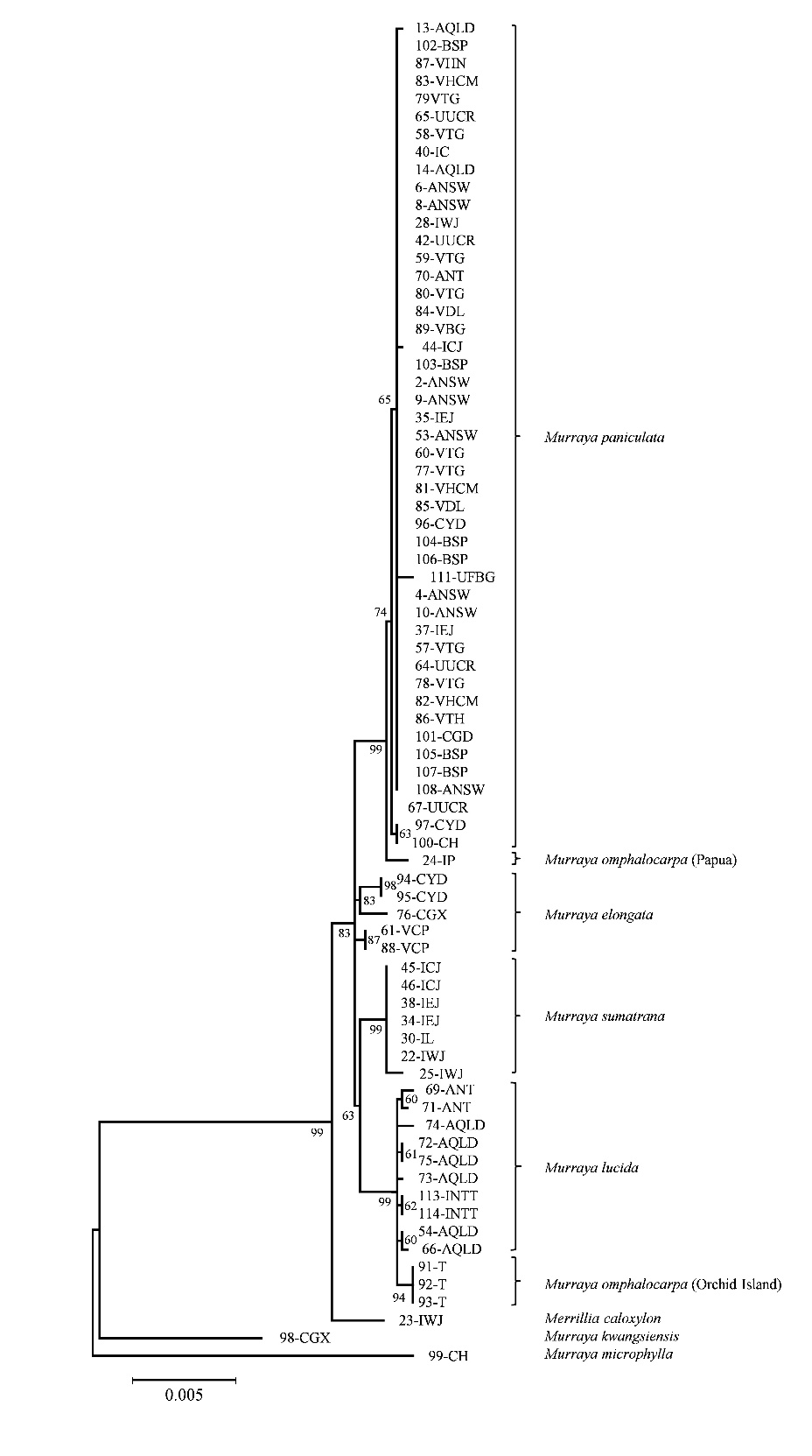


**Figure S1**. Phylogenetic analysis of the combined sequences of six chloroplastal regions from accessions of *Murraya* and *Merrillia*. The evolutionary history was inferred by using maximum likelihood based on the Tamura 3-parameter model (Tamura 1992). The tree with the highest log likelihood (-6747.00) is shown. The percentage of trees in which the associated taxa clustered together is shown next to the branches. A discrete gamma distribution was used to model evolutionary rate differences among sites (5 categories (+G, parameter = 0.3618)). The tree is drawn to scale, with branch lengths measured in the number of substitutions per site.

**
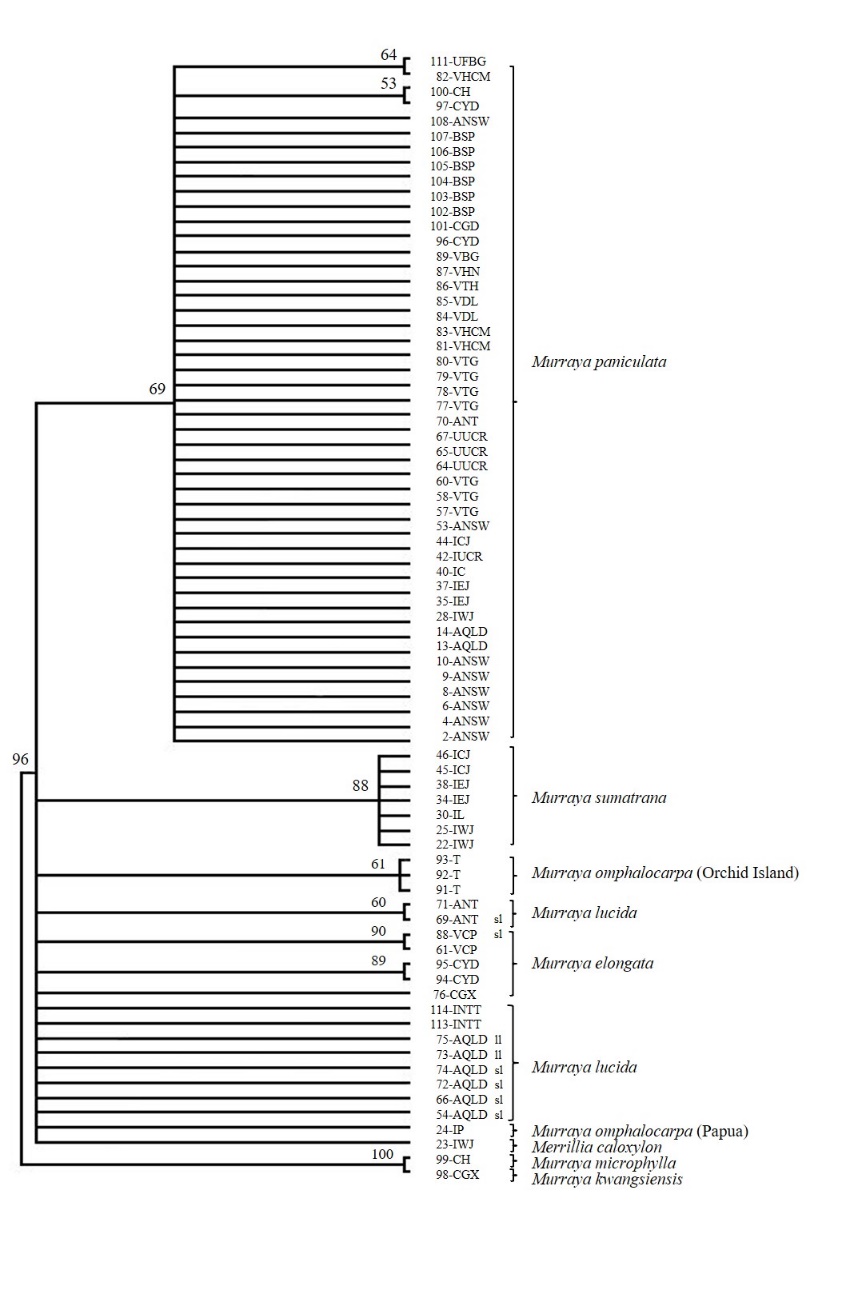
**

**Figure S2.** 50% majority-rule bootstrap consensus tree based on the indels of six chloroplastal regions from accessions of *Murraya* and *Merrillia* derived from maximum parsimony analysis. *Murraya kwangsiensis* and *M. microphylla* were used as the outgroup. Bootstrap values are provided as percentages from 1000 replications. ‘sl’ small leaflet and ‘ll’ large leaflet forms of *Murraya lucida* from Australia.


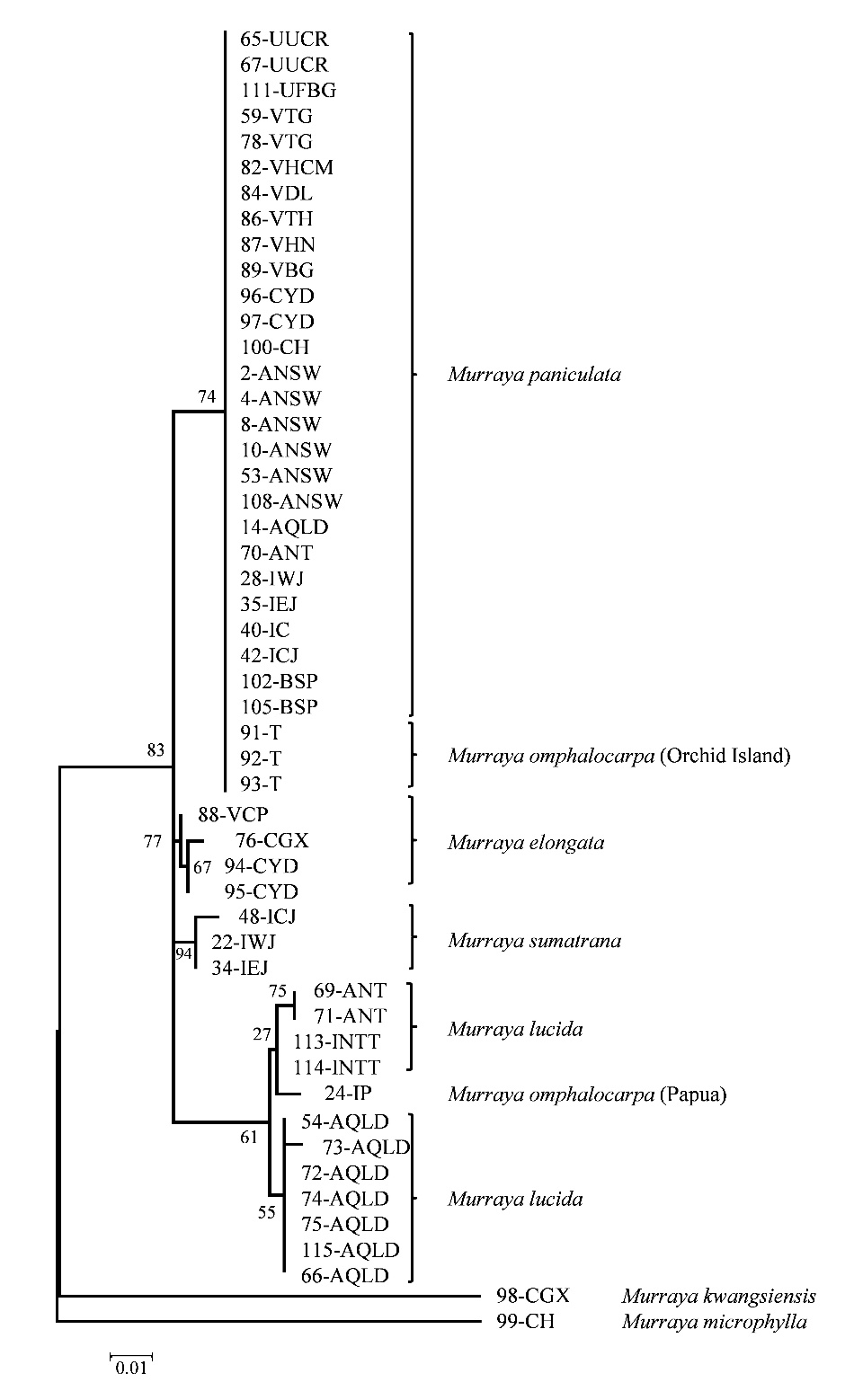


**Figure S3**. Phylogenetic analysis by of the ITS regions of accessions of *Murraya*. The evolutionary history was inferred using the maximum likelihood method based on the Tamura 3-parameter model (Tamura 1992). The tree with the highest log likelihood (-1487.98) is shown. A discrete gamma distribution was used to model evolutionary rate differences among sites (5 categories (+G, parameter = 0.1993)). The tree is drawn to scale, with branch lengths measured in the number of substitutions per site.


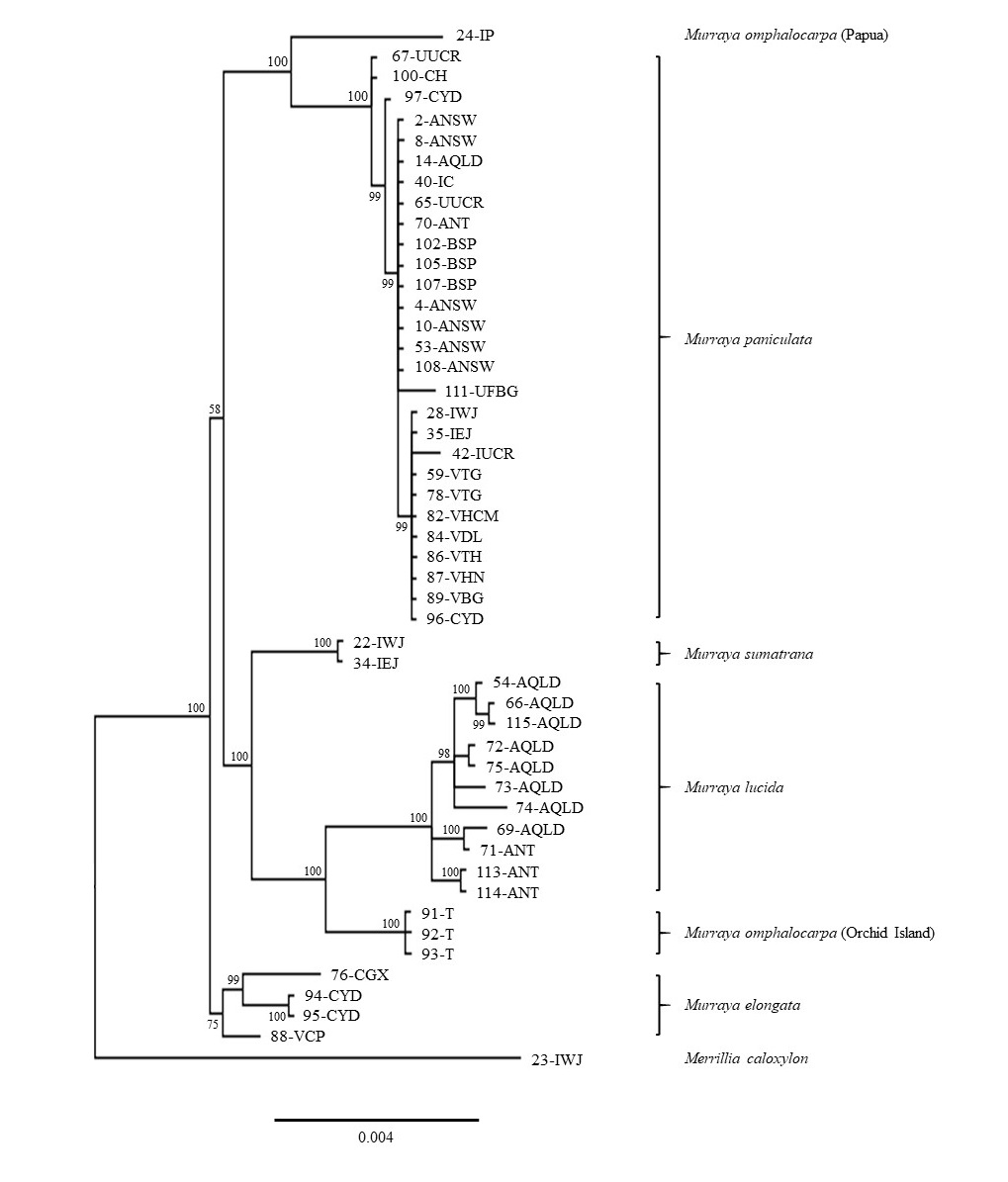


**Figure S4**. Bayesian inference tree based on the 6 chloroplastal regions combined with the ITS region of accessions of *Murraya* and *Merrillia* following partitioning and model selection using PartitionFinder 2 (Lanfear et al. 2016) using the greedy algorthim (Lanfear et al. 2012). The partitioning scheme identified as best was subset 1 (*trnC-ycf6*, *trnL-F*, *psbM-trnD*, *matK-trnK*, *rps4-trnT*, *rps16*); subset 2 (ITS) with GTR (cpDNA) and GTR+G (ITS) substitution models. The Markov chains were run for 1,000,000 generations with a sample frequency of 1000 and a burnin of 250,000 generations. The tree is drawn to scale, with branch lengths measured in the number of substitutions per site and posterior probabilities are shown for each branch.

References

Kubitzki K, Kallunki JA, Duretto MF, Wilson PG: Rutaceae. In: Kubitzki K, editor. The Families and Genera of Flowering Plants. vol. X: Flowering Plants: Eudicots: Sapindales, Cucubitales, Myrtaceae. Berlin, Germany: Springer; 2011: 276-356.

Lanfear R, Calcott B, Ho S, Guindon S. PartitionFinder: combined selection of partitioning schemes and substitution models for phylogenetic analyses. Molecular Biology and Evolution 2012, 29(6): 1695-1701.

Lanfear R, Frandsen PB, Wright AM, Senfeld T, Calcott B PartitionFinder 2: new methods for selecting partitioned models of evolution for molecular and morphological phylogenetic analyses. Molecular Biology and Evolution 2016, 34(3): 772-773.

Swingle WT, Reece CR: The botany of *Citrus* and its wild relatives. In: Reuther W, Webber HJ, Batchelor LD, editors. The Citrus Industry. vol. 1. Berkeley, CA, USA: Division of Agricultural Sciences, University of California; 1967: 190–430.

Tamura K. Estimation of the number of nucleotide substitutions when there are strong transition-transversion and G + C-content biases. Molecular Biology and Evolution 1992, 9(4): 678-687.
